# Comparison of the efficacy based on clinicopathological characteristics and the safety of first-line treatments for patients with advanced ALK rearrangement non-small cell lung cancer: a network meta-analysis

**DOI:** 10.3389/fonc.2025.1620485

**Published:** 2026-01-19

**Authors:** Yanwei Li, Yunxin Wen, Wenjing Zhang, Yurong Zhao, Ligui Zhou, Xianrong Zeng, Xuefeng Kang, Luzhen Li

**Affiliations:** 1 Department of Oncology, Zhongshan Hospital of Traditional Chinese Medicine Affiliated to Guangzhou University of Traditional Chinese Medicine, Zhongshan, Guangdong, China; 2 Department of Obstetrics and Gynecology, Zhongshan Hospital of Traditional Chinese Medicine Affiliated to Guangzhou University of Traditional Chinese Medicine, Zhongshan, Guangdong, China

**Keywords:** ALK, efficacy, first-line, network meta-analysis, non-small cell lung cancer, safety

## Abstract

**Background:**

Despite multiple phase III randomized controlled trials (RCTs) establishing first-line treatments for advanced anaplastic lymphoma kinase (ALK) rearrangement non-small cell lung cancer (NSCLC), the optimal regimen for diverse clinicopathological features remains unclear.

**Methods:**

PubMed, Embase, Cochrane Library, and ClinicalTrials.gov were searched for RCTs. The results of progression-free survival (PFS), overall survival (OS), objective response rate (ORR), grade 3–4 adverse events (AEs), and System Organ Class (SOC)-specific AEs (including hepatic, hematological, and gastrointestinal AEs) were compared and ranked, using network meta-analysis (NMA) and the surface under the cumulative ranking curve (SUCRA), with PFS considering various clinicopathological characteristics.

**Results:**

A total of 3040 participants from 11 RCTs were enrolled, with data encompassing 10 distinct therapeutic regimens. In the overall patient cohort, lorlatinib achieved the longest PFS (93.9%) and the highest ORR (70.1%), whereas alectinib administered at a dose of 600 mg twice daily (bid) conferred the most favorable OS (83.7%) and the lowest incidence of grade 3–4 AEs (87.1%). The PFS efficacy profiles of the 10 regimens exhibited significant heterogeneity stratified by clinicopathological characteristics. Specifically, lorlatinib demonstrated superior efficacy in the Non-Asian subgroup (86.8%), patients without brain metastasis (84.7%), those with Eastern Cooperative Oncology Group performance status (ECOG PS) 0/1 (78.5%), males (71.2%), females (83.9%), patients aged < 65 years (74.3%), and never-smoking patients (89.7%). Alectinib (300 mg bid) demonstrated the optimal efficacy in the subgroups of brain metastasis (83.2%) and smoking history (90%), while alectinib (600 mg bid) ranked first in the subgroups of age ≥ 65 years (73%) and ECOG PS 2 (69.3%). Ensartinib achieved the optimal PFS in the Asian subgroup (71.8%). With respect to SOC-specific AEs, alectinib (300 mg bid) was associated with the lowest risk of hepatic AEs (87%) but carried the highest risk of anemia (11.3%). Iruplinalkib showed the lowest incidence of hematological AEs (72.2%), and alectinib (600 mg bid) had the lowest risk of gastrointestinal AEs (78.6%).

**Conclusions:**

Lorlatinib demonstrated PFS advantage for advanced ALK rearrangement NSCLC, but OS benefit remains unestablished. Alectinib had the lowest hepatic and gastrointestinal AEs risk, while iruplinalkib had the lowest hematological AEs risk.

**Systematic review registration:**

https://www.crd.york.ac.uk/prospero/, identifier CRD42023495527.

## 1 Introduction

Lung cancer is the leading cause of cancer-related mortality worldwide (1), and non-small cell lung cancer (NSCLC) accounts for about 85% of cases (2), and anaplastic lymphoma kinase (ALK) rearrangements occur in about 5% of patients with NSCLC (3). The EML4-ALK fusion gene was first identified in NSCLC patients in 2007 (4). Crizotinib was approved as the therapy for patients with ALK rearrangement locally advanced or metastatic NSCLC by the US FDA in 2011. This approval marked a paradigm shift in the treatment of ALK rearrangements NSCLC, transitioning the therapeutic landscape from sole reliance on conventional chemotherapy. As the first ALK tyrosine kinase inhibitor (TKI), it significantly improved progression-free survival (PFS) compared to chemotherapy (5, 6), thus opening the door for targeted treatment of ALK rearrangement NSCLC. Subsequently, to address resistance issues and achieve better survival benefit, second-generation ALK TKIs such as ceritinib, brigatinib, alectinib, ensartinib, iruplinalkib, and envonalkib were developed, along with the third-generation lorlatinib. All have completed phase III clinical trials, demonstrating a significant improvement in PFS compared to chemotherapy or crizotinib (7–14).

Although multiple first-line treatment options are available, the lack of direct comparisons of their efficacy and safety complicates clinical decision-making. Although previous network meta-analyses have evaluated the efficacy of various regimens in ALK rearrangement NSCLC (15, 16), few studies have comprehensively considered individual clinicopathological features to identify optimal treatment strategies. Although previous network meta-analyses compared system organ class (SOC)-specific adverse events (AEs) (17), the substantial heterogeneity in available SOC-specific AEs across trials led to imprecise results. In contrast, our study compares specific AEs, groups them by SOC, and synthesizes their performance to rank SOC-specific AEs, rather than directly comparing SOC-specific AEs. This approach reduces heterogeneity and improves accuracy. Using all available data, we conducted a network meta-analysis on a wide range of AEs to evaluate the safety of multiple interventions.

In the study, we comprehensively enrolled relevant RCTs and systematically extracted and synthesized clinical data to perform network meta-analysis (NMA), enabling direct and indirect comparisons of the efficacy and safety of 10 treatment regimens. The individualized treatment of NSCLC necessitates a careful balance between the efficacy and toxicity of different regimens. Crucially, NMAs were performed to evaluate treatment efficacy across diverse characteristics, such as brain metastasis, age, ECOG PS, sex, smoking history, and ethnicity, providing evidence to tailor treatment strategies based on patients’ clinicopathological features. Furthermore, NMAs were conducted to compare the safety of various regimens based on grade 3–4 AEs and SOC-specific AEs (e.g., hepatic, hematological, gastrointestinal AEs), providing clinicians with evidence to select treatments with differing toxicity profiles.

## 2 Materials and methods

This NMA was conducted in accordance with the PRISMA guidelines for NMAs (**Supplementary Table S1**) (18). The study was registered in PROSPERO under the identifier CRD42023495527.

### 2.1 Literature search strategy

A comprehensive literature search was conducted across multiple databases, including PubMed, Embase, the Cochrane Central Register of Controlled Trials, and Clinical Trials, without language restrictions, covering records from their inception until December 2, 2024. The specific search strategy is outlined in **Supplementary Table S2**.

### 2.2 Study eligibility and identification

Phase III RCTs were included in the NMA based on predefined criteria. Eligible studies involved patients with histologically confirmed stage III/IV NSCLC harboring ALK rearrangement, compared at least two first-line treatment arms (with at least one arm including ALK-TKIs), and reported at least one clinical benefit, such as PFS, overall survival (OS), objective response rate (ORR), grade 3–4 AEs, specific AEs. Studies were excluded if they focused on maintenance or neoadjuvant therapy, involved interventions such as immunotherapy or radiotherapy, had fewer than 30 patients in any treatment arm, or if they were conference abstracts, brief reports, or lacked safety data. To ensure data quality and avoid redundancy, only the most recent and comprehensive trials were included. In cases where updated data were unavailable, previously reported data were utilized to maintain the integrity of the analysis.

### 2.3 Data extraction and risk of bias assessment

The raw data from the enrolled trials were extracted into a spreadsheet, including study name, publication year, first author, characteristics of patients (e.g., sex, age, smoking history, ethnicity, brain metastasis status, ECOG), interventions, outcomes of endpoints (e.g., PFS, OS, ORR, grade 3–4 AEs, specific AEs). If data assessed by the Independent Review Committee (IRC) are available, they will be used; otherwise, data assessed by investigators will be utilized. The risk of bias in each study was evaluated utilizing the Cochrane Risk of Bias Tool (ROB 2.0) —a widely validated, standardized instrument specifically developed for assessing methodological quality and quantifying potential biases in RCTs (19). Data extraction and bias risk evaluation were performed independently by two researchers (WYX and ZYR). In cases of disagreement, resolution was achieved through discussions.

### 2.4 Statistical analysis

The primary endpoint of this study was PFS, while secondary endpoints included OS, ORR, and grade 3–4 AEs, as well as SOC-specific AEs. Hazard ratios (HRs) and odds ratios (ORs) with their corresponding 95% confidence intervals (CIs) were employed to assess survival outcomes (PFS, OS) and categorical outcomes (ORR, grade 3–4 AEs, and SOC-specific AEs), respectively. We used the deviance information criterion (DIC) to compare consistency and inconsistency models, ultimately selecting the random-effects consistency model based on DIC (**Supplementary Table S3**) results and considerations of NMA reliability. The NMA was performed using a Bayesian approach, employing Markov Chain Monte Carlo (MCMC) simulations. This analysis was carried out with the assistance of the GEMTC and JAGS packages in R software (version 4.3.1). To estimate PFS and OS, the analysis involved 500,000 iterations, including an initial adaptation phase of 50,000 iterations, with a thinning interval set to 1. The computational parameters for other endpoints can be found in (**Supplementary Table S4**). Convergence of the NMAs was assessed through trace plots and the Brooks-Gelman-Rubin statistic. Study heterogeneity was evaluated using Cochran’s Q test and the I² statistic, visualized in forest plots. Significant heterogeneity was defined as an I² value exceeding 50% or a Q test P-value below 0.1. Treatments were ranked using surface under the cumulative ranking curve (SUCRA), with higher scores indicating better efficacy (PFS, OS, ORR) and lower risk for AEs. The SUCRA value (**Supplementary Table S5**) for SOC-specific AEs was calculated as the average of SUCRA value for its corresponding specific AEs.

## 3 Results

### 3.1 Characteristics of the studies and risk of bias

**Figure 1:**
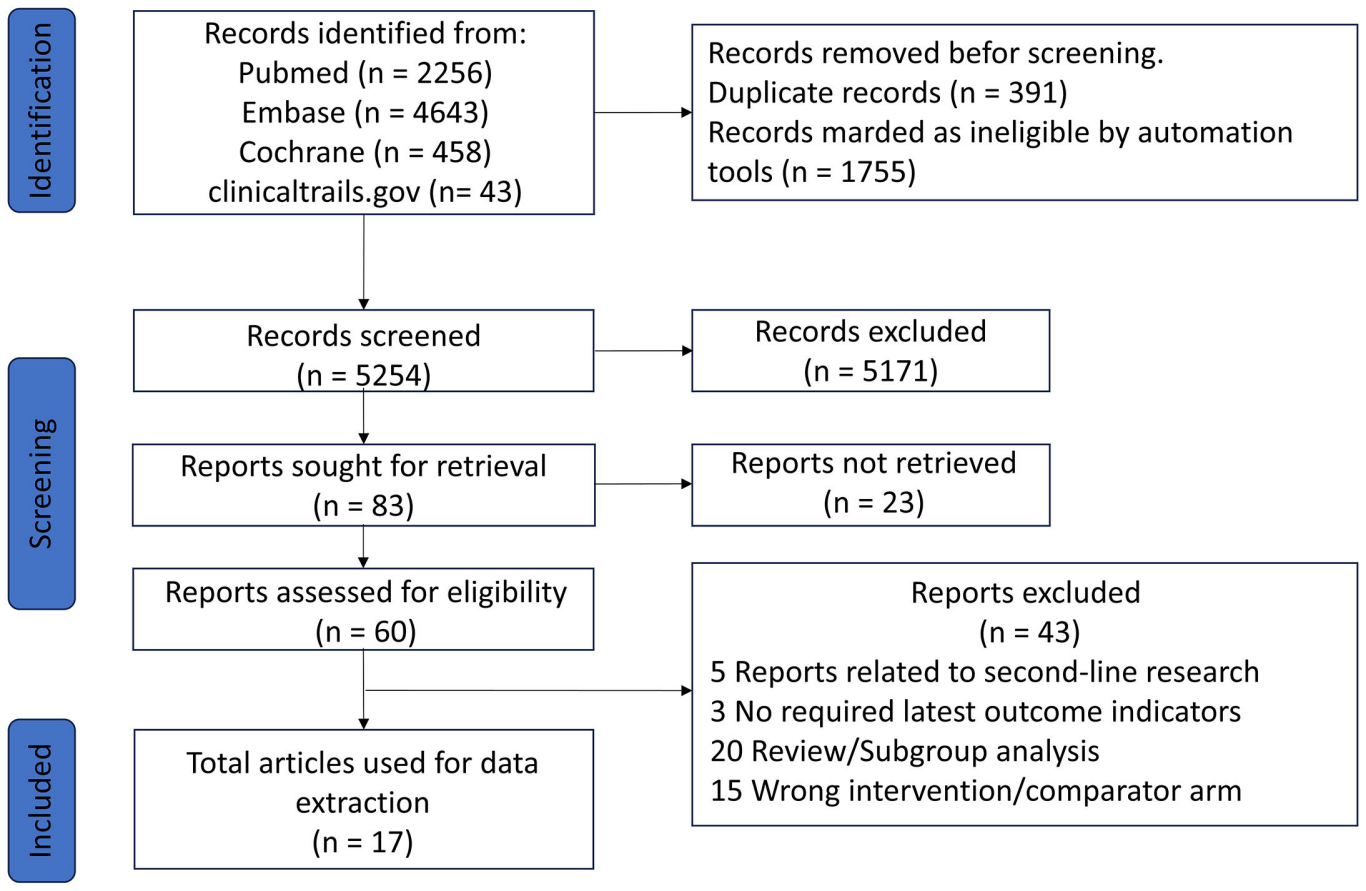
Study flow chart.

The details of the literature screening process are shown in **Figure 1**, and the main characteristics of the RCTs are summarized in **Table 1**. A total of 7,400 records were collected from the databases mentioned. Of these, 7,317 were excluded based on the selection criteria, and 83 studies were selected for full-text review. In the end, 17 articles meeting the eligibility criteria were included in our analysis, which covered 11 phase III RCTs (PROFILE 1014 (20–22), PROFILE 1029 (6), ASCEND-4 (12), ALEX (23, 24), J-ALEX (9), ALESIA (14), ALTA-1L (7, 25), CROWN (26–28), eXalt3 (10), NCT04009317 (13), INSPIRE (11), encompassing a total of 3040 patients and 10 treatment regimens.

**Table 1:** Baseline characteristics of ALK-TKIs trials.

Study	Phase	Sample size	Median age	Sex Female/Male	Region	Intervention arm	Control arm	Reported outcomes
PROFILE 1014 (20–22)	III	172/171	52/54	212/131	Global	Crizotinib 250mg bid	Cisplatin/Carboplatin 75mg/m^2^/3w	PFS; OS; ORR; 3–4 AEs
PROFILE 1029 (6)	III	104/103	48/50	107/100	Asian	Crizotinib 250mg bid	Cisplatin/Carboplatin 75mg/m^2^/3w; Pemetrexed 500mg/m^2^/3w	PFS; OS; ORR; 3–4 AEs
ASCEND-4 (12)	III	189/187	55/54	216/160	Global	Ceritinib 750mg qd	Cisplatin/Carboplatin 75mg/m^2^/3w; Pemetrexed 500mg/m²/3w	PFS; OS; ORR; 3–4 AEs
ALEX (24)	III	152/151	56/54	171/132	Global	Alectinib 600mg bid	Crizotinib 250mg bid	PFS; OS; ORR; 3–4 AEs
J-ALEX (9)	III	103/104	61/60	125/82	Asian	Alectinib 300mg bid	Crizotinib 250mg bid	PFS; OS; ORR;
ALESIA (14)	III	125/62	51/49	109/98	Asian	Alectinib 600mg bid	Crizotinib 250mg bid	PFS; OS; ORR; 3–4 AEs
ALTA-1L (7, 25)	III	137/138	58/60	150/125	Global	Brigatinib 180mg qd	Crizotinib 250mg bid	PFS; OS; ORR; 3–4 AEs
CROWN (26–28)	III	149/147	61/56	175/121	Global	Lorlatinib 100mg qd	Crizotinib 250mg bid	PFS; OS; ORR; 3–4 AEs
eXalt3 (10)	III	143/147	54/53	141/149	Global	Ensartinib 225mg qd	Crizotinib 250mg bid	PFS; OS; ORR; 3–4 AEs
NCT04009317 (13)	III	131/133	53/52	128/136	Asian	Envonalkib 600mg bid	Crizotinib 250mg bid	PFS; ORR; 3–4 AEs
INSPIRE (11)	III	143/149	55/55	158/134	Asian	Iruplinalkib 180mg qd	Crizotinib 250mg bid	PFS; OS; ORR;

The assessment of ROB is presented in **Supplementary Figure S1**. Overall, the ROB in all RCT studies was generally low. However, in PROFILE 1014, ASCEND-4, ALEX, PROFILE 1029, and CROWN, more participants withdrew from the control group than the experimental group, leading to a “Some Concerns” rating for “Deviations from Intended Interventions.” Besides, the evaluation of patient-reported outcomes (PROs) is subjective, and patients were aware of their interventions, resulting in a “Some Concerns” rating for “Measurement of the Outcome”.

### 3.2 Network meta-analysis in overall patients

**Figure 2:**
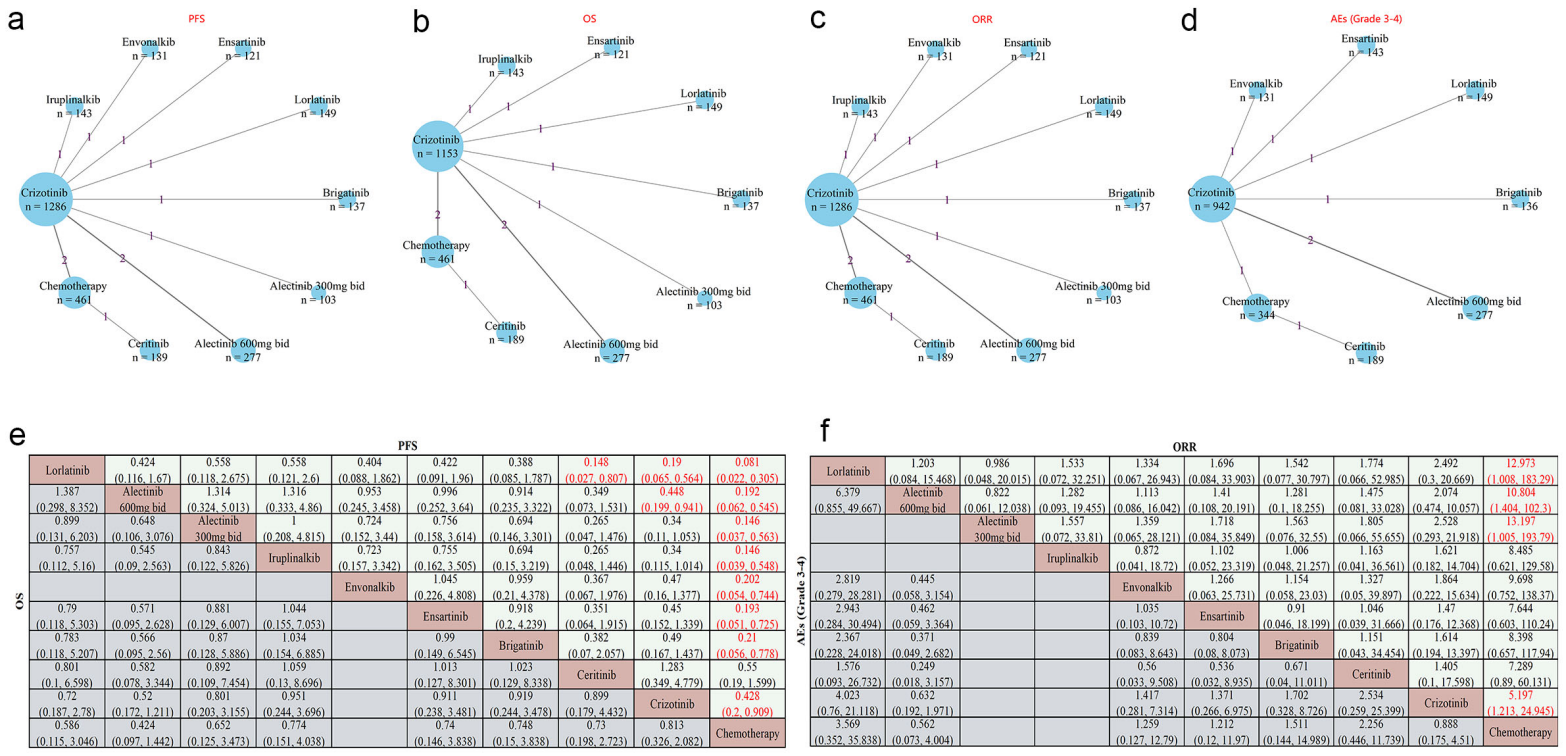
Network meta-analysis of PFS, OS, ORR, and Grade 3–4 AEs in advanced ALK rearrangement NSCLC patients **(a–d)**. Network diagrams show treatments as nodes and head-to-head comparisons as lines. Node size is based on the number of patients receiving the treatment, and line thickness is based on the number of head-to-head studies. Numbers on lines indicate direct comparison studies. **(e)** Pooled HR (95% CI) for PFS and OS. **(f)** Pooled OR (95% CI) for ORR and Grade 3–4 AEs. Each cell presents HR or OR for row *vs*. column treatments; HR < 1 always favors the row treatment. OR > 1 favors the row treatment in ORR, while OR < 1 in grade 3–4 AEs indicates that the row treatment has a lower incidence of AEs. Significant results are highlighted in red.

**Figures 2a, b** showcase the network plots for PFS and OS that encompass all participants from the included randomized controlled trials (RCTs), with additional network plots available in **Supplementary Figure S2**. In terms of PFS, all TKIs-related regimens, except ceritinib, were superior to chemotherapy (HR < 1 and P < 0.05) (**Figure 2e**). Besides, lorlatinib provided significant advantages compared with crizotinib (HR 0.19, 95% CI 0.065–0.564, P < 0.05) and ceritinib (HR 0.148, 95% Cl 0.027–0.807, P < 0.05) (**Figure 2e**). Additionally, alectinib (600mg bid) exhibited modest superiority over crizotinib (HR 0.448, 95% Cl 0.199–0.941, P < 0.05). Regarding OS, no significant differences were observed across the 9 regimens. Notably, alectinib (600 mg bid) was associated with HRs < 1 compared with all other treatments (**Figure 2e**), though none of these comparisons reached statistical significance.

**Figures 2c, d** illustrate the network plots for ORR and grade 3–4 AEs, respectively, which were subsequently compared among various regimens based on data from 11 RCTs (for ORR) and 8 RCTs (for grade 3–4 AEs), as shown in **Figure 2f**. Alectinib, crizotinib, and lorlatinib demonstrated significantly higher objective response rates (ORR) compared to chemotherapy (OR > 1 and all P < 0.05). For grade 3–4 adverse events, lorlatinib showed ORs > 1 and alectinib (600 mg bid) showed ORs < 1 versus other treatments (**Figure 2f**), though neither pattern was statistically significant.

### 3.3 PFS subgroup analysis based on various clinicopathological characteristics

#### 3.3.1 Sex, age, smoking

**Figure 3:**
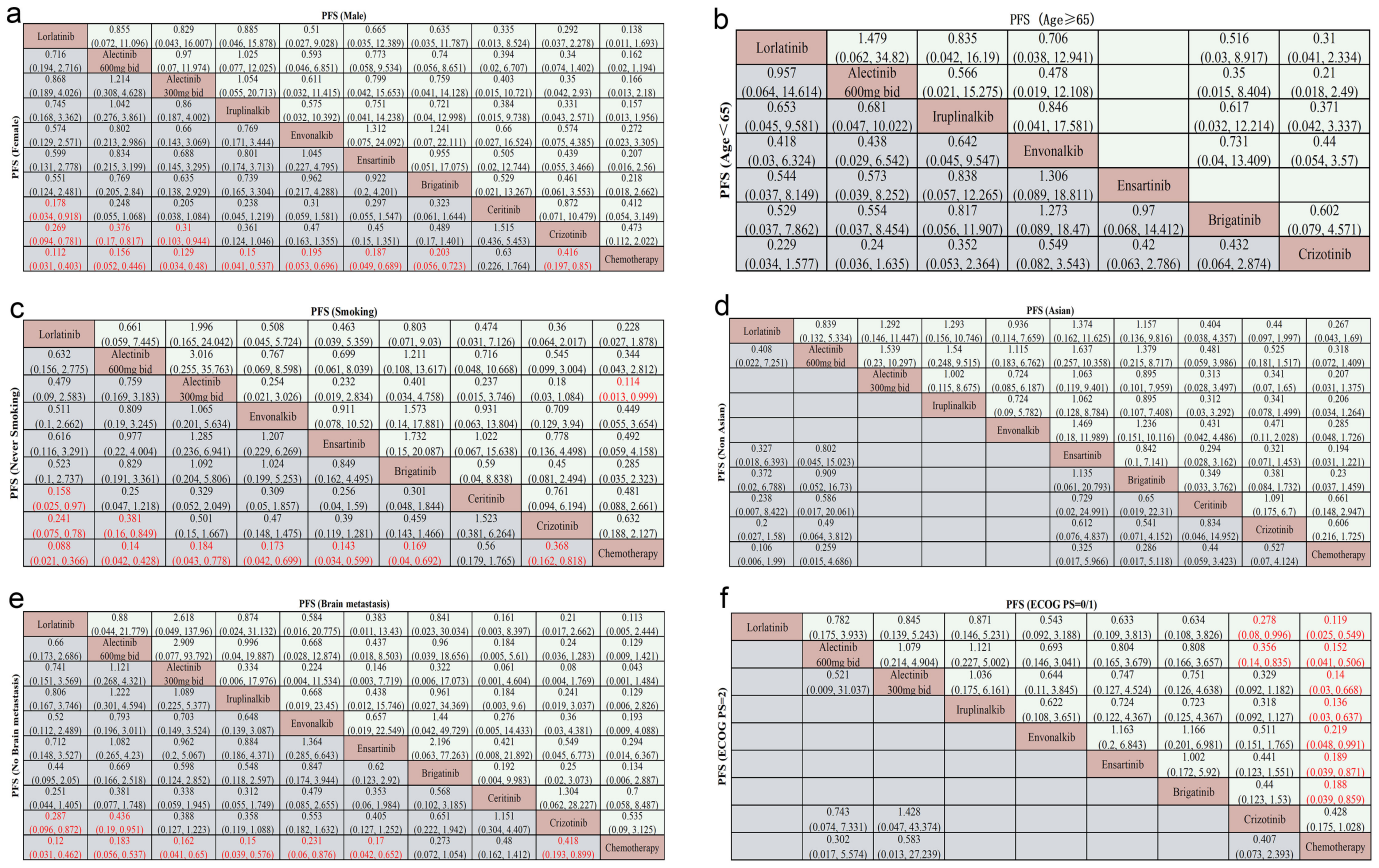
Subgroup network meta-analysis of PFS in patients with advanced ALK rearrangement NSCLC, categorized by clinical characteristics. Pooled hazard ratios (HRs) with 95% CIs are shown for **(a)** gender (male *vs*. female), **(b)** age (≥65 *vs*. <65 years), **(c)** smoking history (smoking *vs*. never smoking), **(d)** ethnicity (Asian *vs*. Non-Asian), **(e)** brain metastases (present *vs*. absent), and **(f)** ECOG performance status (PS = 0/1 *vs*. PS = 2). Significant results are highlighted in red.

In addressing the variable of gender, the findings indicate that, for females, all TKIs regimens, with the exception of ceritinib, demonstrated superior efficacy compared to chemotherapy (HR < 1 and P < 0.05) (**Figure 3a**). Notably, lorlatinib conferred significant advantages over crizotinib (HR 0.269, 95% CI 0.094–0.781, P < 0.05) and ceritinib (HR 0.178, 95% CI 0.034–0.918, P < 0.05) (**Figure 3a**). Furthermore, compared with crizotinib, alectinib (600mg HR 0.376, 95% CI 0.17–0.817 P < 0.05) and alectinib (300 mg bid) (HR 0.31, 95% CI 0.103–0.944; P < 0.05), provided superior efficiency in PFS (**Figure 3a**). Although HRs in males favored lorlatinib (all HR < 1), the results were not statistically significant (**Figure 3a**).

In the age-based subgroup analysis (<65 *vs* ≥65 years), lorlatinib showed HRs <1 for all comparisons in younger patients, and alectinib (600 mg bid) showed HRs <1 for all comparisons in older patients (**Figure 3b**); however, none were statistically significant.

8 regimens, with the exception of ceritinib, were superior to chemotherapy regarding PFS in the never-smoking subgroup (HR < 1, P < 0.05), while fewer was observed in the smoking subgroup. In never-smoking subgroup, lorlatinib significantly outperformed crizotinib (HR 0.241, 95% CI 0.075–0.78, P < 0.05) (**Figure 3c**) and ceritinib (HR 0.158, 95% CI 0.025–0.97, P < 0.05) (**Figure 3c**). Alectinib (600 mg bid) also showed moderate superiority over crizotinib (HR 0.381, 95% CI 0.16–0.849, P < 0.05) (**Figure 3c**). While in smoking patients, alectinib (300 mg bid) exhibited moderate advantage over chemotherapy (HR 0.114, 95%CI 0.013, 0.999, P < 0.05) (**Figure 3c**). All other TKI regimens showed HRs < 1 versus chemotherapy (**Figure 3c**), although none reached statistical significance.

#### 3.3.2 Ethnicity, brain metastasis, ECOG

In the Asian subgroup (10 regimens) and the non-Asian subgroup (7 regimens), ensartinib was associated with HRs < 1 compared with all other regimens in Asians, and lorlatinib showed HRs < 1 versus all other regimens in non-Asians (**Figure 3d**). However, none of these comparisons reached statistical significance.

In patients without brain metastasis, all TKIs regimens, with the exception of ceritinib and brigatinib, were superior to chemotherapy (HR < 1, P < 0.05) (**Figure 3e**). Above all, compared with crizotinib, lorlatinib (HR 0.287, 95% CI 0.096–0.872, P < 0.05) (**Figure 3e**) and alectinib (600 mg bid) (HR 0.436, 95% CI 0.19–0.951, P < 0.05) (**Figure 3e**), provided superior efficiency in PFS. In patients with brain metastases, all TKIs had HRs < 1 compared with chemotherapy, and alectinib (300mg bid) had HRs < 1 versus all other regimens. However, none of these comparisons reached statistical significance.

The ECOG PS = 0/1 subgroup has 9 regimens, while the PS = 2 subgroup has 3 regimens. In the ECOG PS = 0/1 subgroup, almost all TKIs regimens, with the exception of crizotinib, were superior to chemotherapy (HR < 1, P < 0.05) (**Figure 3f**). Above all, compared with crizotinib, lorlatinib (HR 0.278, 95% CI 0.08–0.996, P < 0.05) (**Figure 3f**) and alectinib (600 mg bid) (HR 0.356, 95% CI 0.14–0.835, P < 0.05) (**Figure 3f**), provided superior efficiency in PFS. In the ECOG PS = 2 subgroup, alectinib (600 mg bid) had HRs < 1 compared with all other regimens (**Figure 3f**), but none of these comparisons reached statistical significance.

### 3.4 Subgroup analysis based on various AEs

**Figure 4:**
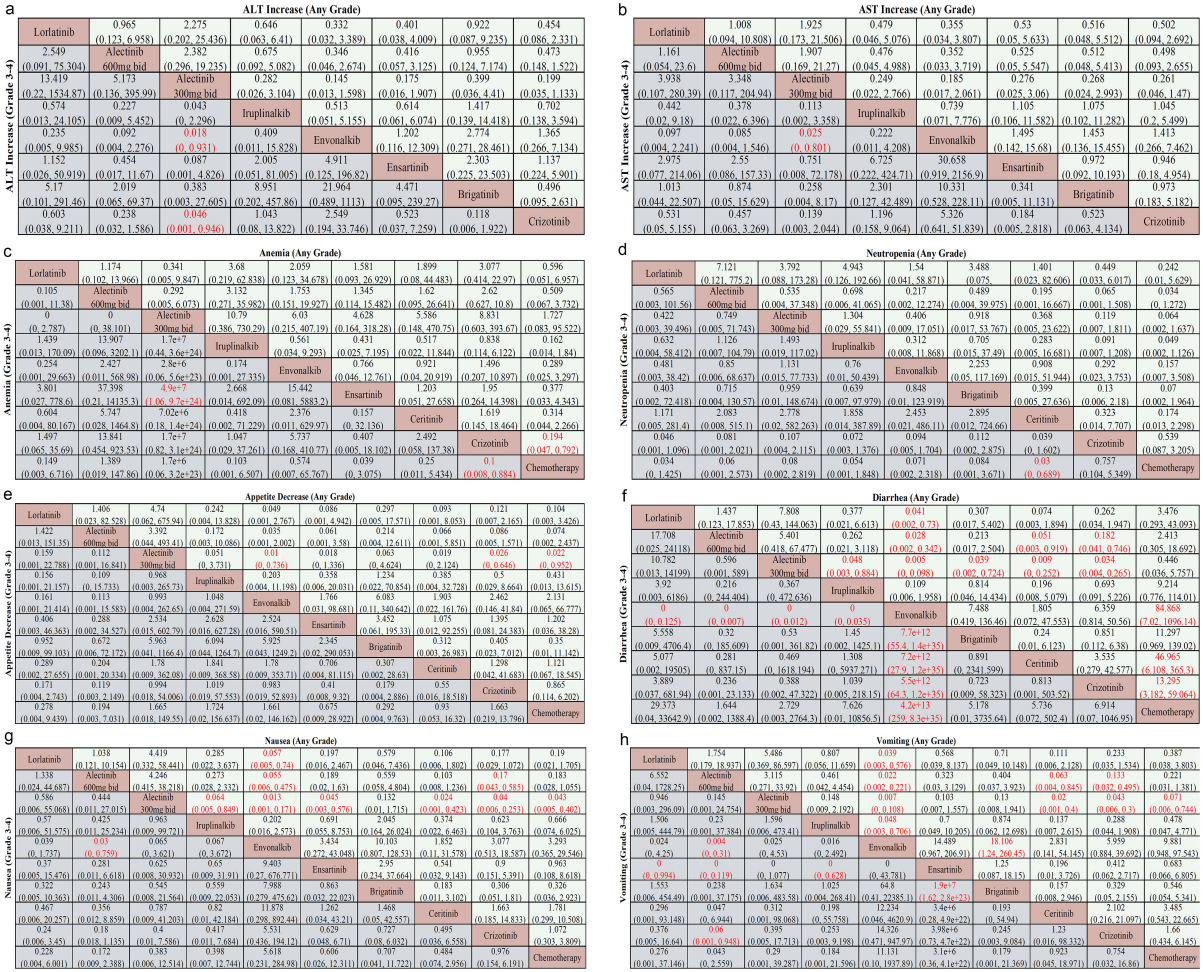
Subgroup network meta-analysis of AEs in patients with advanced ALK rearrangement NSCLC. Pooled odds ratios (ORs) with 95% CIs are shown for **(a)** ALT increases (any grade *vs*. grade 3-4), **(b)** AST increases (any grade *vs*. grade 3-4), **(c)** anemia (any grade *vs*. grade 3-4), **(d)** neutropenia (any grade *vs*. grade 3-4), **(e)** appetite decrease (any grade *vs*. grade 3-4), **(f)** diarrhea (any grade *vs*. grade 3-4), **(g)** nausea (any grade *vs*. grade 3-4), and **(h)** vomiting (any grade *vs*. grade 3-4). Significant results are highlighted in red.

In the subgroup with increased ALT or AST, alectinib (300 mg bid) was associated with HRs < 1 for safety-related endpoints (**Figures 4a, b**), although none of these comparisons reached statistical significance. Notably, it was safer than envonalkib (HR 0.018, 95% CI 0–0.931, P < 0.05) and crizotinib (HR 0.046, 95% CI 0.001–0.946, P < 0.05) in the grade 3–4 Increased ALT subgroup (**Figure 4a**). Similar safety benefit was observed against envonalkib in the grade 3–4 Increased AST subgroup (HR 0.025, 95% CI 0–0.801, P < 0.05) (**Figure 4b**). Conversely, envonalkib was associated with HRs > 1 for hepatotoxicity-related events in both subgroups (**Figures 4a, b**), though none of these comparisons reached statistical significance.

In the anemia subgroup, alectinib (300 mg bid) appeared to be the least safe regimen (all HR > 1) (**Figure 4c**), particularly when compared to ensartinib for grade 3–4 anemia (HR > 1, P < 0.05) (**Figure 4c**). Crizotinib demonstrated significant safety in both any grade anemia (HR 0.194, 95% CI 0.047–0.792, P < 0.05) and grade 3–4 anemia subgroups (HR 0.1, 95% CI 0.008–0.884, P < 0.05) compared to chemotherapy (**Figure 4c**). Conversely, in the analysis of anemia, iruplinalkib showed HRs < 1 for any-grade events and ensartinib showed HRs < 1 for grade 3–4 events compared with other regimens (**Figure 4c**). However, none of these comparisons reached statistical significance.

In the analysis of neutropenia, chemotherapy was associated with HRs > 1 for AEs compared with all other regimens (**Figure 4d**), while alectinib (600 mg bid) showed HRs < 1 versus all comparators (**Figure 4d**); none of these comparisons reached statistical significance. For grade 3–4 neutropenia, ceritinib also had HRs < 1 compared with all other regimens (**Figure 4d**), with a statistically significant reduction in risk versus chemotherapy (HR 0.03, 95% CI 0–0.689; P < 0.05).

In the analysis of appetite decrease, alectinib (300 mg bid) was associated with HRs < 1 for any-grade events compared with all other regimens (**Figure 4e**), with statistically significant reductions versus envonalkib (HR 0.01, 95% CI 0–0.736; P < 0.05), crizotinib (HR 0.26, 95% CI 0–0.646; P < 0.05), and chemotherapy (HR 0.22, 95% CI 0–0.952; P < 0.05). For grade 3–4 appetite decrease, alectinib (600 mg bid) also showed HRs < 1 versus all comparators (**Figure 4e**). Additionally, envonalkib and iruplinalkib were each associated with HRs < 1 across all comparisons for any-grade and grade 3–4 appetite decrease, respectively (**Figure 4e**); however, these latter comparisons did not reach statistical significance.

Envonalkib was the least safe for grade 3–4 diarrhea (all HR > 1, P < 0.05) (**Figure 4f**) and also underperformed compared to lorlatinib, both doses of alectinib, and chemotherapy for any grade diarrhea (all HR > 1, P < 0.05) (**Figure 4f**). In contrast, for grade 3–4 diarrhea, chemotherapy was associated with HRs < 1 compared with all other regimens (**Figure 4f**), though these comparisons did not reach statistical significance. Alectinib (300mg bid) demonstrated the lowest risk among iruplinalkib, envonalkib, brigatinib, ceritinib, and crizotinib in the any grade diarrhea subgroup, with HRs of 0.048, 0.005, 0.039, 0.009, and 0.034, respectively (all P < 0.05) (**Figure 4f**).

In the nausea subgroup, alectinib (300mg bid) showed better safety compared to iruplinalkib, envonalkib, ensartinib, ceritinib, crizotinib, and chemotherapy (all HR < 1, P < 0.05) (**Figure 4g**) for any grade of nausea. Alectinib (600mg bid) also demonstrated superior safety compared to envonalkib for both grade 3–4 nausea (HR 0.03, 95% CI 0–0.759, P < 0.05) (**Figure 4g**) and any-grade nausea (HR 0.055, 95% CI 0.006–0.475, P < 0.05) (**Figure 4g**), as well as to crizotinib for any-grade nausea (HR 0.17, 95% CI 0.043–0.585, P < 0.05) (**Figure 4g**). Conversely, envonalkib was less safe than lorlatinib and both doses of alectinib (all HR > 1, P < 0.05) (**Figure 4g**) for any grade of nausea, and inferior to alectinib (600mg bid) for grade 3–4 nausea (HR > 1, P < 0.05) (**Figure 4g**).

In the vomiting subgroup, alectinib (300mg bid) demonstrated a significantly lower risk of any-grade vomiting compared to envonalkib, ceritinib, crizotinib, and chemotherapy (all HR < 1, P < 0.05) (**Figure 4h**). Alectinib (600mg bid) showed a significantly lower risk of grade 3–4 vomiting than envonalkib, ensartinib, crizotinib (all HR < 1, P < 0.05) (**Figure 4h**). Conversely, most other regimens, particularly lorlatinib, alectinib (600mg bid), iruplinalkib, and brigatinib (all HR < 1, P < 0.05) (**Figure 4h**), provided better safety for grade 3–4 vomiting compared to ensartinib. Similarly, against envonalkib, the majority of other treatments, especially lorlatinib, both doses of alectinib, and brigatinib (all HR < 1, P < 0.05) (**Figure 4h**), offered improved safety for any grade of vomiting.

## 4 Rank probabilities

**Figure 5:**
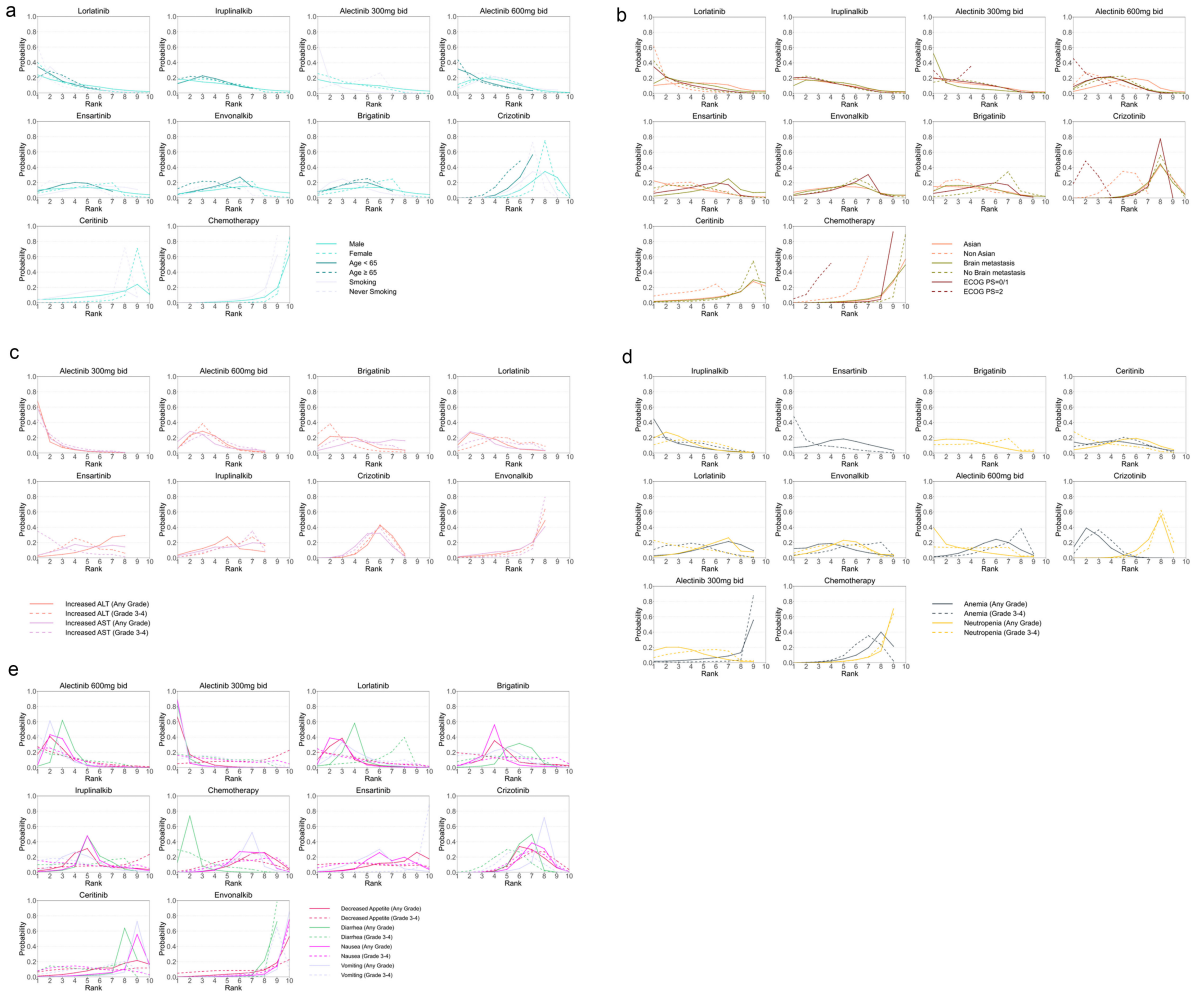
Bayesian ranking profiles for treatments concerning PFS in patients with advanced ALK-mutant NSCLC, categorized by clinicopathological features. **(a)** PFS based on sex, age, and smoking history. **(b)** PFS based on ethnicity, brain metastasis status, and ECOG PS. **(c)** SOC-specific AEs: hepatic AEs (increased ALT/AST). **(d)** SOC-specific AEs: hematological AEs (anemia/neutropenia). **(e)** SOC-specific AEs: gastrointestinal AEs (decreased appetite, diarrhea, nausea, vomiting). These profiles show each treatment’s ranking likelihood: first to last (best to worst) for PFS, and lowest to highest risk for SOC-specific AEs.

**Figure 6:**
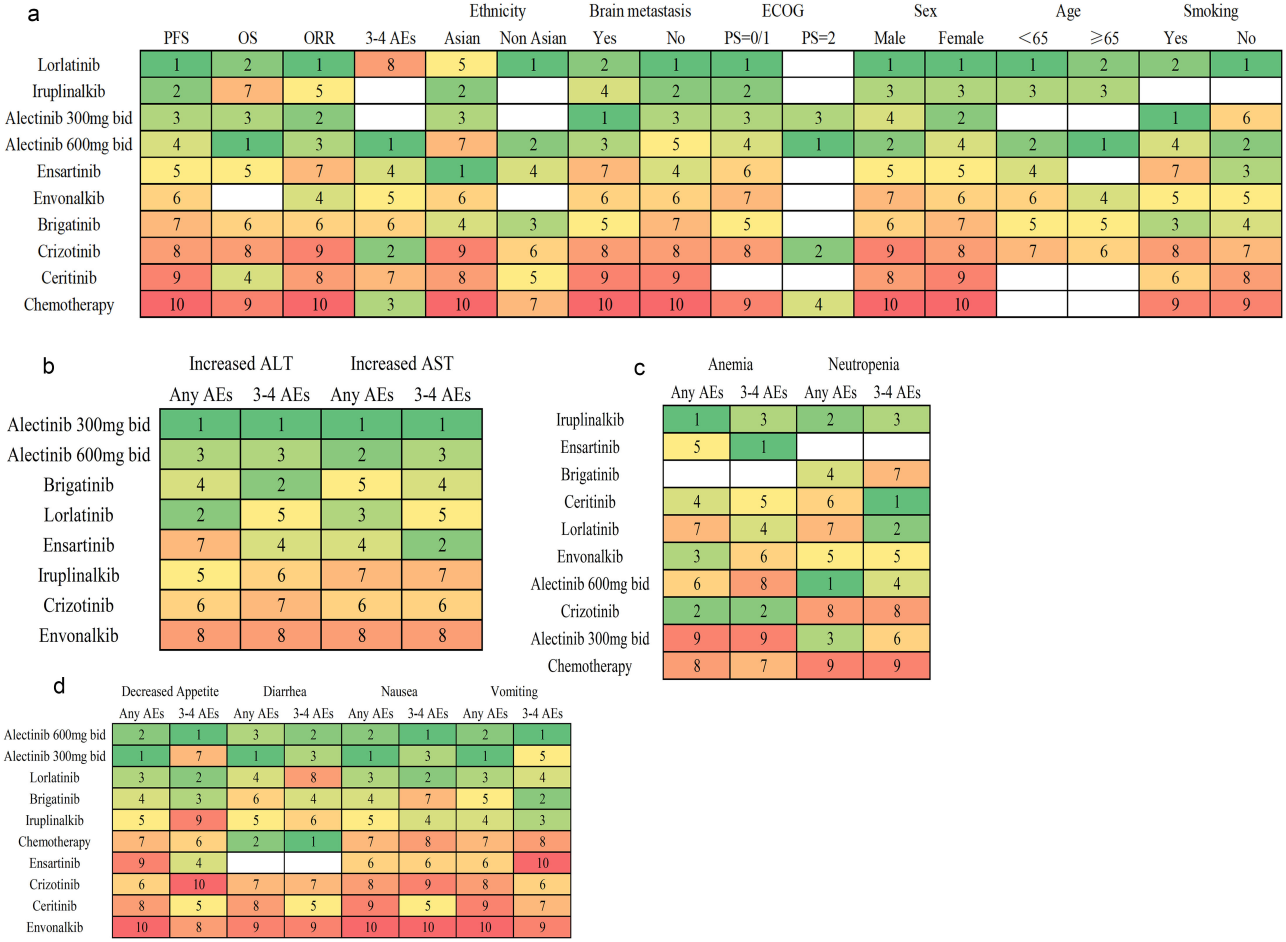
Cumulative probability ranking of treatments for advanced ALK rearrangement NSCLC patients, evaluating: **(a)** efficacy (PFS, OS, ORR, subgroup PFS) and overall grade 3–4 AEs; **(b)** hepatic AEs (any grade and grade 3–4 increased ALT/AST); **(c)** hematological AEs (any grade and grade 3–4 anemia/neutropenia); and **(d)** gastrointestinal AEs (any grade and grade 3–4 decreased appetite, diarrhea, nausea, vomiting). The first rank represents the highest efficacy or the lowest risk of AEs.

The Bayesian ranking probabilities and SUCRA rank for various regimens are presented in **Figures 5**, **6**. These cover overall patients, PFS and AEs and their subgroups, as well as OS and ORR, and are generally consistent with the NMA. For overall patients, lorlatinib led in PFS (93.9%) and ORR (70.1%) but had the highest risk of 3–4 AEs (13.2%). Alectinib (600mg bid) excelled in OS (83.7%) and had the lowest risk of 3–4 AEs (87.1%). Various regimens were evaluated for PFS across different clinicopathological characteristics. Lorlatinib emerged as the top-ranked treatment for subgroups including Non-Asians (86.8%), patients without brain metastasis (84.7%), those with ECOG PS = 0/1 (78.5%), all genders (male 71.2%, female 83.9%), individuals < 65 (74.3%), and never-smoking (89.7%). Conversely, alectinib (300mg bid) was most effective for those with brain metastasis (83.2%) and smoking (90%), while alectinib (600mg bid) led in patients with ECOG PS = 2 (69.3%) and those age ≥ 65 (73%). Ensartinib was the leading choice for the Asian subgroup (71.8%). The cumulative probability ranking curves can be found in **Supplementary Figure S3**.

Next, various regimens were ranked based on SOC-specific AEs including hepatic, hematological, and gastrointestinal AEs. Hepatic AEs were assessed by elevated ALT and AST levels, hematological AEs by anemia and neutropenia, and gastrointestinal AEs by appetite loss, diarrhea, nausea, and vomiting, all evaluated across all grades and specifically grades 3-4. Alectinib (300mg bid) had the lowest risk in hepatic AEs (87%), alectinib (600mg bid) in gastrointestinal AEs (78.6%), whereas envonalkib posed the highest in both (14.4%, 11.3%, respectively). For hematological AEs, iruplinalkib presented the least risk (72.2%), while chemotherapy posed the highest risk for neutropenia (7.4%), and alectinib (300 mg bid) for anemia (11.3%).

## 5 Heterogeneity assessment

Forest plots illustrating paired efficacy comparisons, accompanied by heterogeneity assessments, are displayed in **Supplementary Figures S4**–**S9**. Our analysis revealed that approximately three-fourths of the comparisons across multiple outcomes exhibited minimal (I^2^ = 0%) or low heterogeneity within the overall population. The consistency model generally outperformed the inconsistency model in terms of fit, as detailed in **Supplementary Table S3**. Additionally, trace plots and Brooks-Gelman-Rubin diagnostic plots in **Supplementary Figures S10**–**S21** confirmed the stable convergence of the model.

## 6 Discussion

Currently, several regimens, including first-, second-, and third-generation TKIs, are approved as initial treatments for NSCLC patients with ALK-sensitive mutations. However, the lack of direct comparisons regarding their efficacy and safety poses significant challenges for clinicians in selecting the most suitable first-line treatment. In our study, we analyzed data from 11 RCTs to determine the best initial therapy for advanced NSCLC with ALK rearrangement. As far as we know, our NMA included the most comprehensive set of RCTs and was the pioneering study to explore the best first-line regimens for advanced NSCLC patients with ALK-sensitive mutations, taking into account clinicopathological characteristics and SOC-specific AEs including hepatic, hematological, and gastrointestinal AEs.

In ALK rearrangement patients, lorlatinib shows superior PFS and ORR, possibly due to its efficient brain penetration and enhanced kinase selectivity compared to other TKIs (29), a result consistent with Zheng’s finding (30). Conversely, alectinib (600mg bid) led the rankings in terms of OS; however, no significant differences were observed in pairwise comparisons, which is inconsistent with Zhao’s findings (16). This may be Zhao’s finding analyzed with the RMST model. Nonetheless, the existing data does not definitively establish alectinib’s superiority in OS. Since the OS data for most regimens are not yet mature (median OS has not been reached), the available 5-year OS rates are 76% for lorlatinib (31) and 62.5% for alectinib (24). Concerning the grade 3–4 AEs, with data for alectinib (300mg bid) being unavailable, alectinib (600mg bid) could be the option with the lowest risk, while lorlatinib might carry the highest, which agrees with the findings of Luo (17). Lorlatinib posed the highest risk of grade 3–4 AEs, notably hyperlipidemia, weight gain, and hypertension (28), though insufficient data precluded a Bayesian NMA for these events.

Advanced NSCLC is often linked to brain metastases, which have a substantial impact on prognosis (32). In a meta-analysis study by (33), it was found that 34.9% of ALK rearrangement patients have brain metastases at diagnosis, with an annual incidence of 0.17 (95% CI 0.10–0.27) for new brain metastases over a median follow-up of 24 months. Alectinib (300mg bid) ranked first in terms of PFS among patients with baseline brain metastasis, consistent with the findings of Chuang and Ma (34, 35). However, Filetti and Zhao concluded that lorlatinib ranked first in PFS among these patients (16, 36). This discrepancy may be due to different effect sizes used. Conversely, lorlatinib led the rankings in terms of patients without brain metastasis. Alectinib and lorlatinib inhibits brain metastasis in lung cancer by reducing the expression of proteins associated with epithelial-mesenchymal transition (EMT) and matrix metalloproteinases (MMPs) (37). It’s important to acknowledge that the data for alectinib (300mg bid) (J-ALEX trial) is exclusively derived from Asian populations, hence, racial and genetic variables should be taken into account when interpreting the findings.

Indeed, the selection of a treatment plan by healthcare providers should take into account significant variables such as age and ECOG PS. Furthermore, research has indicated that both the side effects and the effectiveness of TKIs may differ based on a person’s age or ECOG PS (11, 23); however, there is a lack of head-to-head and indirect clinical trials to directly validate this perspective. Firstly, in term of age, lorlatinib ranked first for patients with age<65, while alectinib (600mg bid) was first for age≥65. Secondly, in term of ECOG PS, studies have shown that ECOG PS is linked to the survival of ALK-positive NSCLC patients with brain metastases (38). Our study assessed the efficacy of each regimen in patients with ECOG PS = 0/1 and ECOG PS = 2. Lorlatinib could be the preferred choice for patients with ECOG PS = 0/1, ranked first in terms of PFS. Nevertheless, alectinib (600mg bid) held the top position for those with an ECOG PS = 2. However, only four clinical trials included ECOG PS = 2 patients with accessible data, covering four treatments. Notably, alectinib (600mg bid) may shows greater PFS benefit in ECOG PS = 2 patients compared to those with ECOG PS = 0/1. For instance, the HR of alectinib (600mg bid) versus crizotinib is 0.356 for PS = 2 and 0.743 for PS = 0/1; similarly, the HR against chemotherapy is 0.152 for PS = 2 compared to 0.302 for PS = 0/1.

At present, factors such as smoking, sex, and ethnicity have been identified as influencing the effectiveness of anti-tumor treatments, including targeted therapies (39–41). Regarding smoking, individuals who smoke exhibit a significantly higher mutational burden compared to non-smokers, with the TP53 mutation being particularly relevant in lung cancer pathology (42). In ALK-positive NSCLC, a substantial prevalence of TP53 co-mutations is associated with an markedly adverse prognosis (43). Lorlatinib and alectinib (300mg bid) were the most effective regimens in extending PFS for the never smoking and the smoking, respectively, aligning with the findings of Lin (39). Moreover, excluding alectinib (300mg bid) and ceritinib, other TKIs may demonstrate better performance in the never smoking compared to the smoking (e.g., HR of lorlatinib versus chemotherapy in never-smoking *vs* smoking: 0.088 *vs* 0.228). Regarding sex, lorlatinib ranked first for both females and males. Furthermore, in the treatment with ALK inhibitors, the benefit for patients of both sexes is similar (40). Conversely, our study found that except for ceritinib, other TKIs may perform better in females compared to males (e.g., HR of lorlatinib versus ceritinib in females *vs* males: 0.178 *vs* 0.335). The precise mechanism remains uncertain and requires further validation. Regarding ethnicity, our study identified ensartinib as the leading treatment in the Asian subgroup, whereas lorlatinib ranked first in the Non-Asian subgroup, aligning with prior research (44–46). Conversely, Li’s study (15) favored iruplinalkib, Zhao’s preferred alectinib (16), and Tao’s (47) supported brigatinib as the best first-line ALK TKI for Asian patients. The observed discrepancy may arise from conducting separate analyses for the two alectinib doses, using different effect sizes, or incorporating updated clinical data.

AEs are vital considerations for clinicians when choosing a treatment regimen. This study employs a Bayesian NMA to evaluate the safety of first-line treatments for ALK-positive NSCLC regarding SOC-specific AEs including the hepatic, hematologic, and gastrointestinal AEs. Concerning hepatic AEs, alectinib (300mg bid) had the lowest risk of increased ALT/AST (any grade and grades 3-4), while envonalkib had the highest, contrasting Tao’s findings of minimal impact with lorlatinib and maximal impact with ceritinib (48). This difference may arise from updated data or methodological variations, as Tao’s study ranked outcomes by incidence, while this study used OR for NMA and SUCRA for ranking. Concerning hematological AEs, focusing on any grade and grades 3–4 anemia and neutropenia, iruplinalkib posed the lowest risk, while chemotherapy the highest risk for neutropenia, alectinib (300 mg bid) the highest risk for anemia. This may be associated with alectinib’s link to the early development of ubiquitous acanthocytosis and extravascular hemolysis (49). Alectinib (300 mg bid) unexpectedly ranked higher for grade 3–4 anemia than alectinib (600 mg bid), potentially attributed to the exclusively Asian population in the J-ALEX trial. Due to the lack of direct comparisons, this should be interpreted with caution. Concerning gastrointestinal AEs, focusing on decreased appetite, diarrhea, nausea, and vomiting across any grade and grades 3-4, alectinib (600 mg bid) demonstrated the lowest risk, while envonalkib showed the highest risk.

The AE profiles of ALK-TKIs are shaped by differences in molecular structure, kinase selectivity, and off-target effects. Alectinib’s benzo[b]carbazole backbone confers high ALK selectivity, minimizing off-target activity and contributing to its lower risk of hepatic and gastrointestinal toxicities (51–53). However, the 300 mg twice-daily dose conferred no safety advantage over the 600 mg regimen—potentially due to differences in follow-up duration and a higher rate of prior chemotherapy (36% in J-ALEX), which may have amplified toxicity. In contrast, crizotinib ranks highest for neutropenia among ALK-TKIs, likely owing to off-target inhibition of c-KIT (Kd ≈ 150–300 nM), a kinase critical for granulopoiesis (54). Ceritinib exhibits elevated gastrointestinal toxicity, possibly through inhibition of insulin-like growth factor 1 receptor (IGF-1R), which is expressed in gastrointestinal tissues (55 ); notably, the 450 mg dose taken with food achieves comparable exposure but a more favorable gastrointestinal safety profile than the 750 mg fasted regimen in ALK-positive NSCLC (56). Finally, envonalkib ranks highest for both gastrointestinal and hepatic AEs (higher rank indicating greater risk), though its underlying pharmacological mechanisms remain undefined due to a lack of published data.

This work contributes several valuable insights. Firstly, we are the first to systematically demonstrate that the efficacy of various regimens differs based on clinicopathological characteristics of patients with ALK rearrangement NSCLC and to rank these regimens within each subgroup. Secondly, we creatively employed a method to analyze the toxicity profiles of regimens: performing network meta-analysis on specific AEs, categorizing them by SOC, averaging SUCRA value within each category, and ranking SOC-specific AEs. Compared to previous studies (17) analyzing toxicity profiles, our method yields results with lower heterogeneity and greater clarity.

Considering the clinicopathological factors such as brain metastasis status, age, ECOG PS, smoking history, sex, and ethnicity, these can significantly impact the effectiveness of various treatment regimens. In our study, we identified the most effective treatment options tailored to different patient profiles. However, it is often the case that patients may not be able to endure the ideal treatment approach in a clinical setting. Therefore, we ranked the grade 3–4 AEs and SOC-specific AEs of each regimen, offering suitable alternative choices for those who cannot tolerate the preferred treatment.

Our study does have certain limitations. Firstly, the number of clinical trials containing the same head-to-head comparison is only two (e.g., only ALEX and ALESIA for alectinib (600 mg bid) *vs*. crizotinib, and only PROFILE 1014 and PROFILE 1029 for crizotinib *vs*. chemotherapy). In such cases, the results of heterogeneity analysis may not be reliable (50). Secondly, the duration of follow-up varied among the trials, leading to some outcomes being based on immature data. For example, lorlatinib has not reached a median PFS after 5 years of follow-up (28), while ensartinib has a follow-up duration of only 36 months (10). Thirdly, clinical trials vary in patient selection, study design, and efficacy assessment, including brain metastasis rates, treatment-naïve status, crossover allowance, and IRC versus investigator evaluations. Fourth, crossover in the control group can attenuate observed overall survival (OS) differences; trials such as ALEX, J-ALEX, ALESIA, CROWN, eXalt3, NCT04009317, and INSPIRE prohibited post-progression crossover, whereas PROFILE 1014, PROFILE 1029, ASCEND-4, and ALTA-1L permitted it—potentially placing the experimental arm at a relative disadvantage in OS assessment. These inherent differences inevitably influence the outcomes and are difficult to quantify, necessitating cautious interpretation of the results. Future research should aim to address these limitations by incorporating larger datasets with longer follow-up durations and standardized trial designs. Additionally, real-world evidence could complement clinical trial data to provide a more comprehensive understanding of treatment outcomes in diverse patient populations.

## 7 Conclusion

Among the various treatment options, lorlatinib demonstrates PFS advantage but OS benefit remains unestablished. Specifically, lorlatinib was likely to be the best first-line regimens for patients in subgroups of Non-Asian, no brain metastasis, ECOG PS = 0/1, male and female, <65, never smoking, alectinib (600mg bid) for subgroup of ECOG PS = 2, ≥65, alectinib (300mg bid) for subgroup of brain metastasis, smoking, ensartinib for subgroup of Asian, respectively. Regarding safety, alectinib (600 mg bid) may represent the safest treatment regimen, while lorlatinib could pose the highest risk. In detail, specifically, alectinib (300 mg bid) had the lowest hepatic AEs risk, alectinib (600 mg bid) the lowest gastrointestinal AEs risk, and envonalkib the highest risk for both. In terms of hematological AEs, iruplinalkib had the lowest risk, while alectinib (300 mg bid) was linked to the highest risk of anemia. In summary, the NMAs offered valuable insights for clinicians to identify the most suitable regimens for advanced ALK mutated NSCLC patients based on their specific clinicopathological features, as well as appropriate alternative options for those who may not tolerate the optimal treatments.

## Data Availability

The original contributions presented in the study are included in the article/**Supplementary Material**. Further inquiries can be directed to the corresponding author.
