# Supplementary material for: Comparison of the efficacy based on clinicopathological characteristics and the safety of first-line treatments for patients with advanced ALK rearrangement non-small cell lung cancer: a network meta-analysis
Source: Front Oncol. 2026 Jan 19;15:1620485. doi: 10.3389/fonc.2025.1620485 (PMC12861906; doi:10.3389/fonc.2025.1620485)
Supplement: Supplementary file 2 [file DataSheet2.docx]

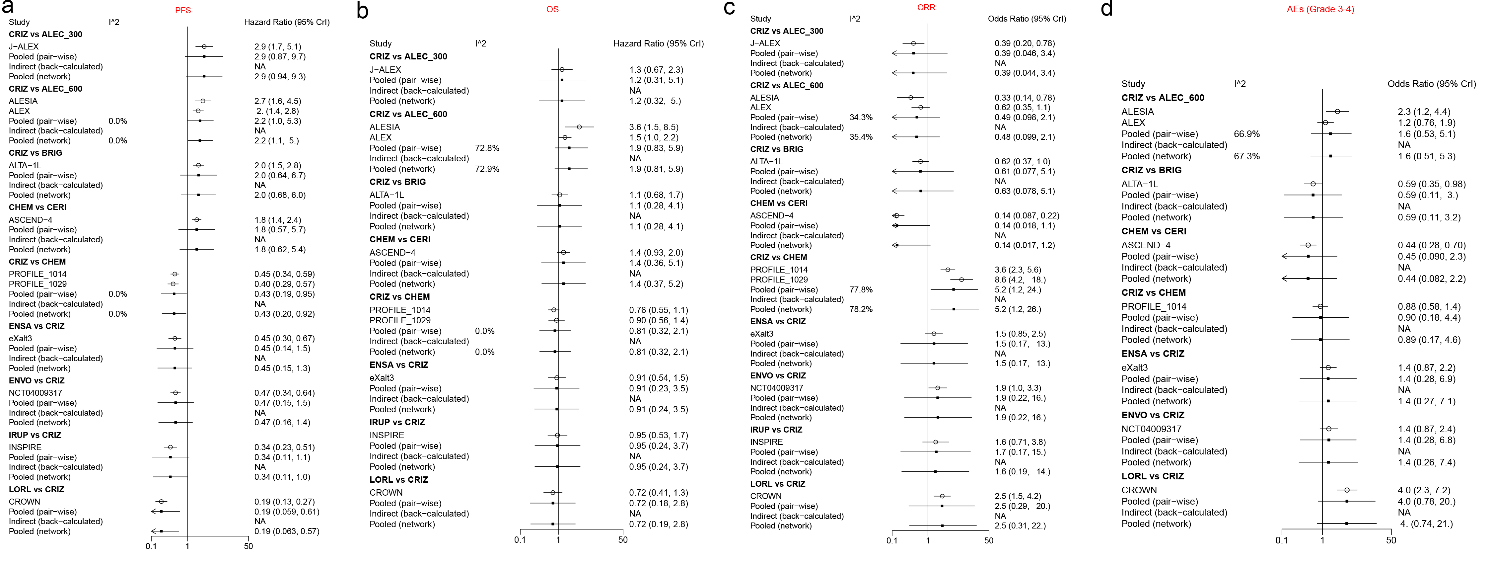


**Fig S4** Heterogeneity analysis for PFS, OS, ORR and AEs (Grade 3-4).


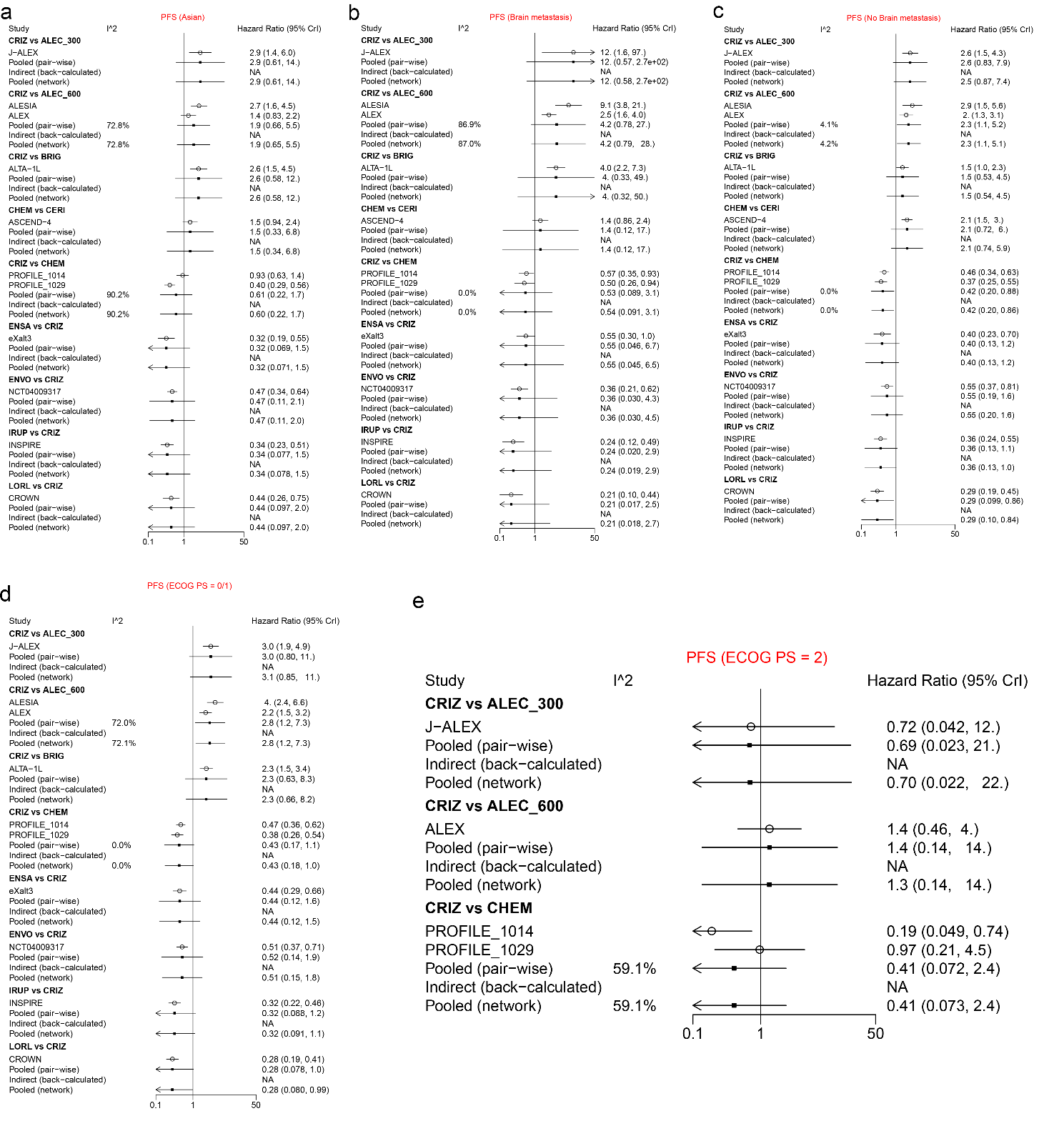


**Fig S5** Heterogeneity analysis for PFS (ethnicity), PFS (brain metastases) and PFS (ECOG PS).


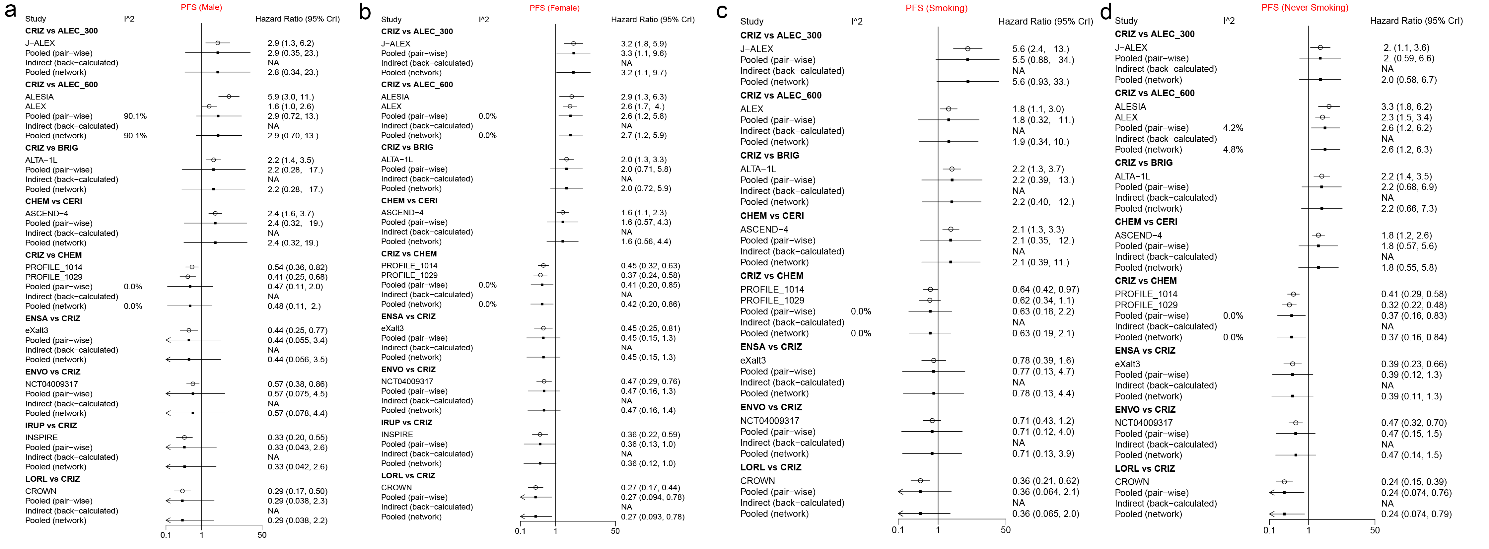


**Fig S6** Heterogeneity analysis for PFS (sex), PFS (age) and PFS (smoking history).


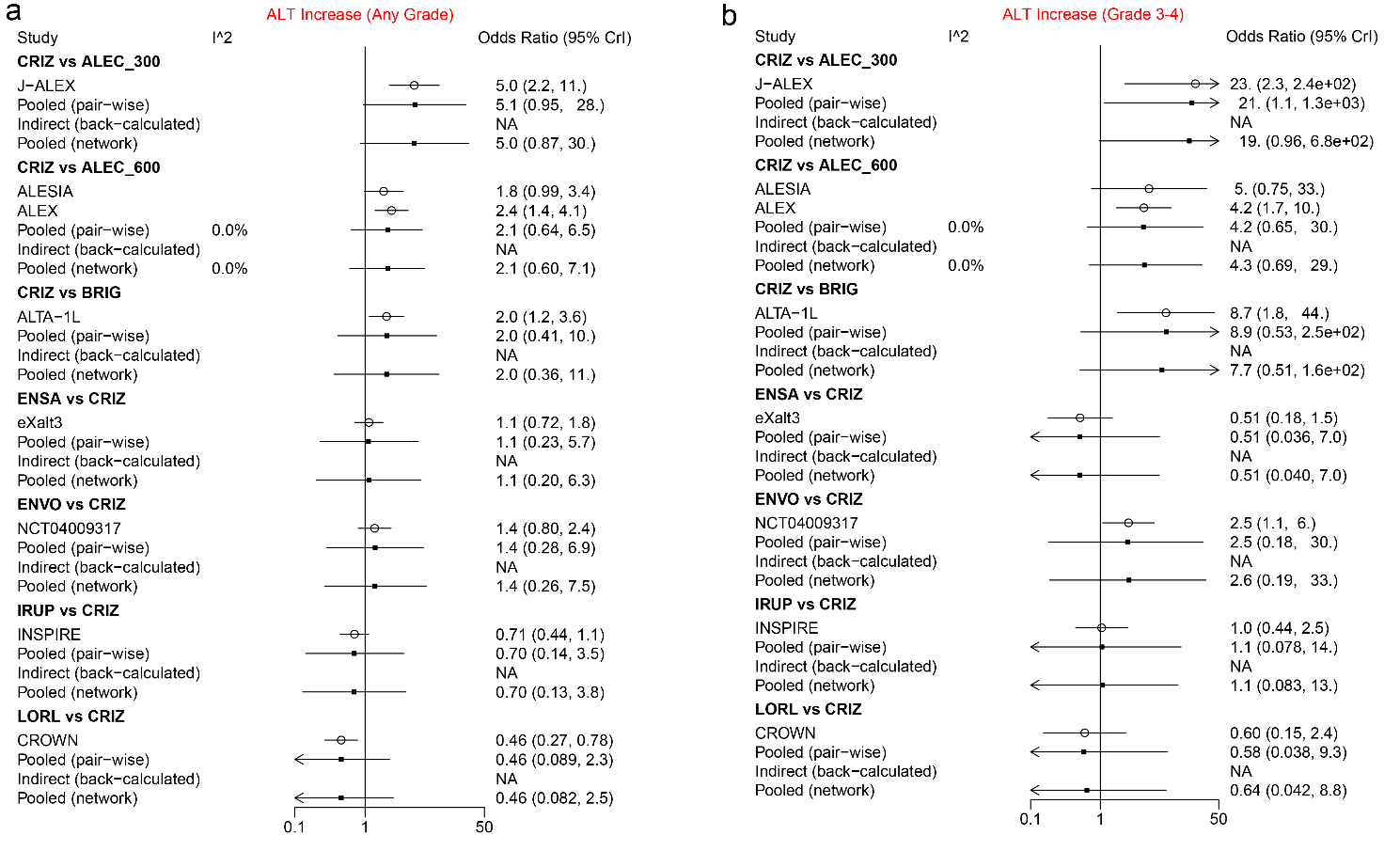


**Fig S7** Heterogeneity analysis for hepatic AEs (increased ALT/AST).


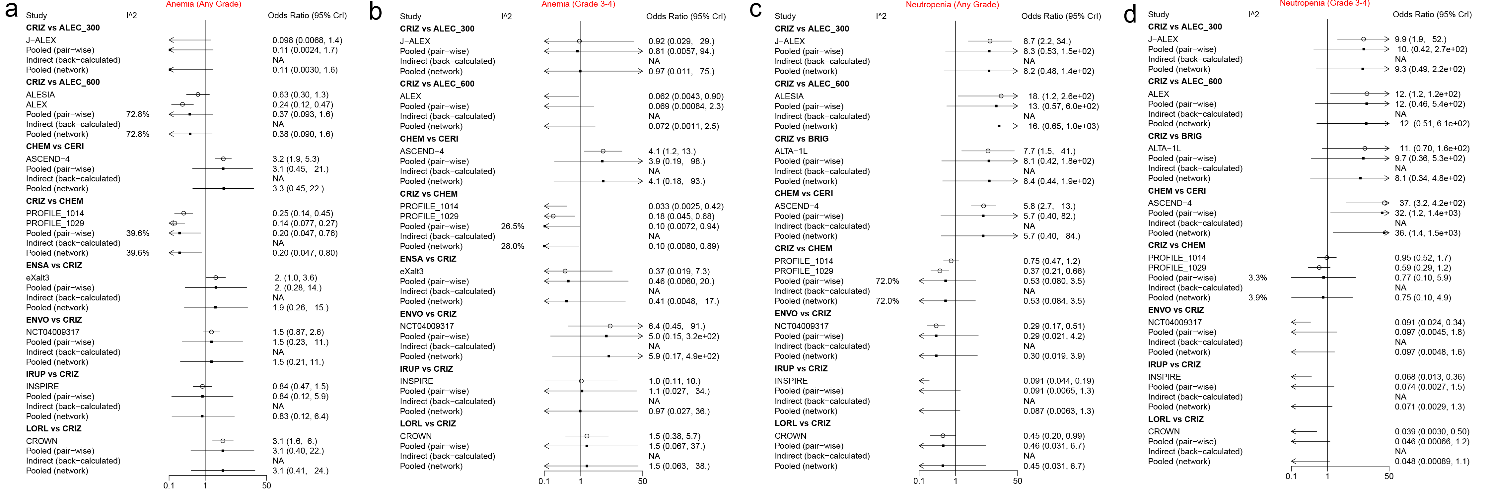


**Fig S8** Heterogeneity analysis for hematological AEs (anemia/neutropenia).


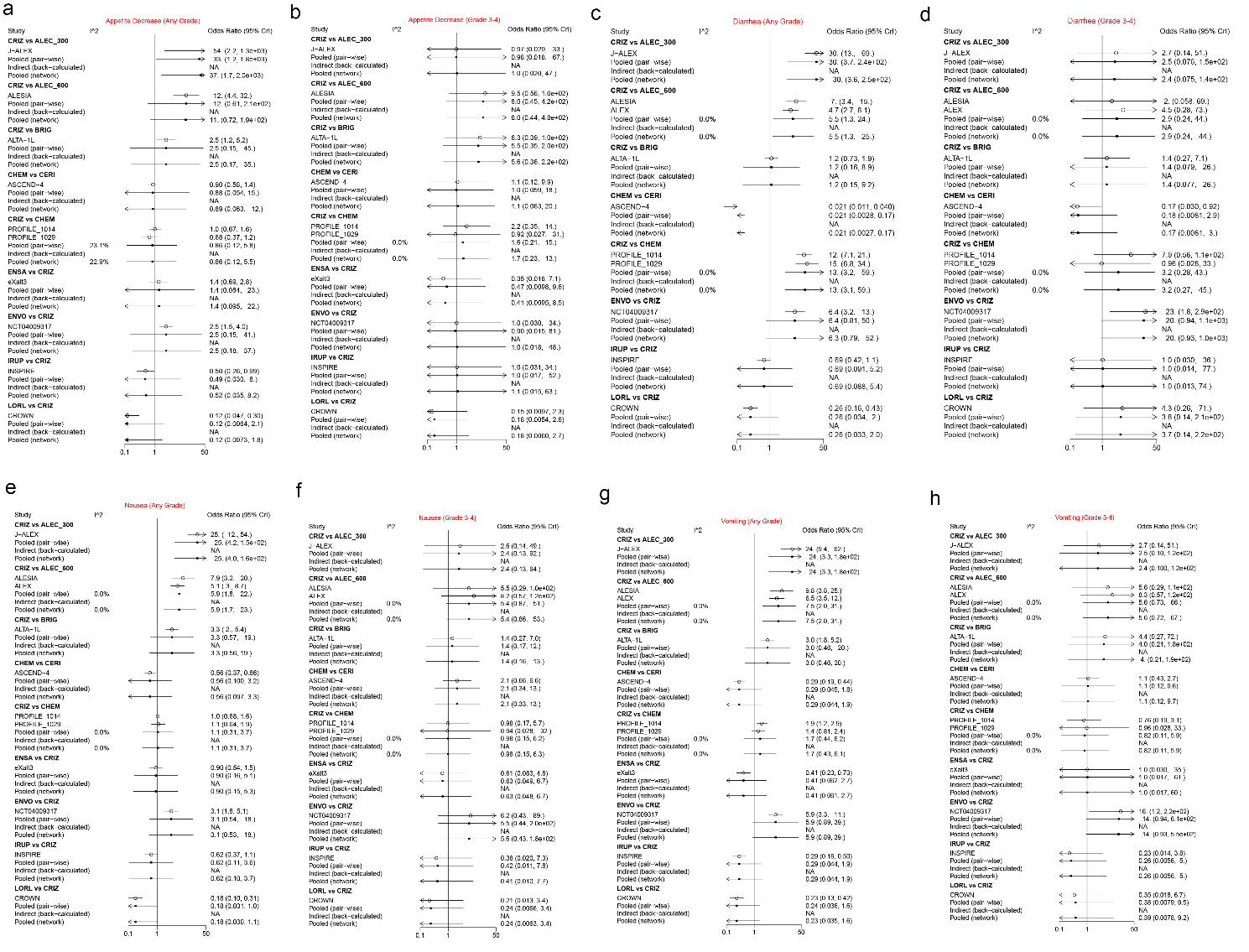


**Fig S9** Heterogeneity analysis for gastrointestinal AEs (decreased appetite, diarrhea, nausea, vomiting).
