# Supplementary material for: Comparison of the efficacy based on clinicopathological characteristics and the safety of first-line treatments for patients with advanced ALK rearrangement non-small cell lung cancer: a network meta-analysis
Source: Front Oncol. 2026 Jan 19;15:1620485. doi: 10.3389/fonc.2025.1620485 (PMC12861906; doi:10.3389/fonc.2025.1620485)
Supplement: Supplementary file 4 [file DataSheet4.docx]

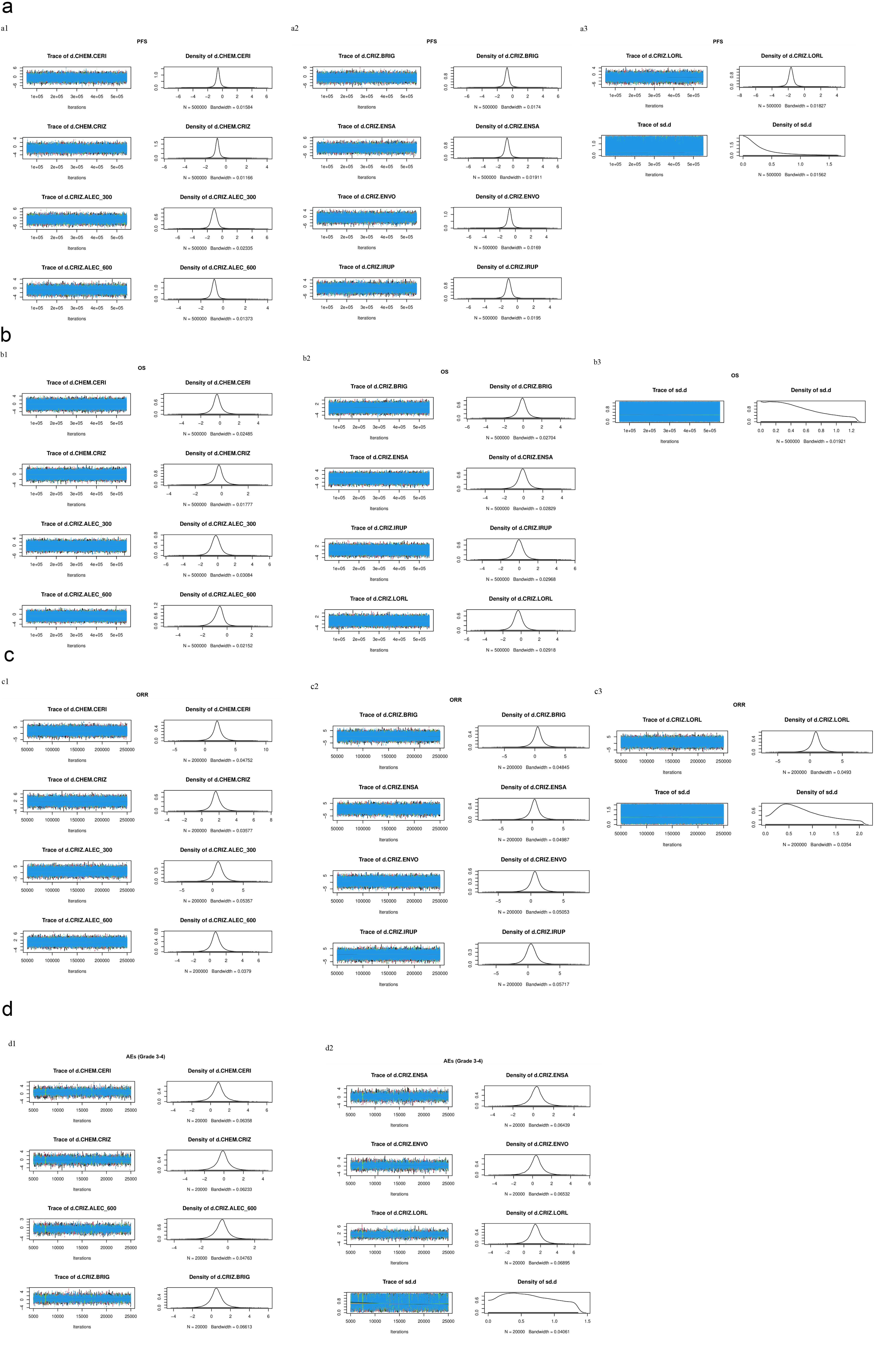


**Fig S16** Trace and Density plot for PFS, OS, ORR and AEs (Grade 3-4).


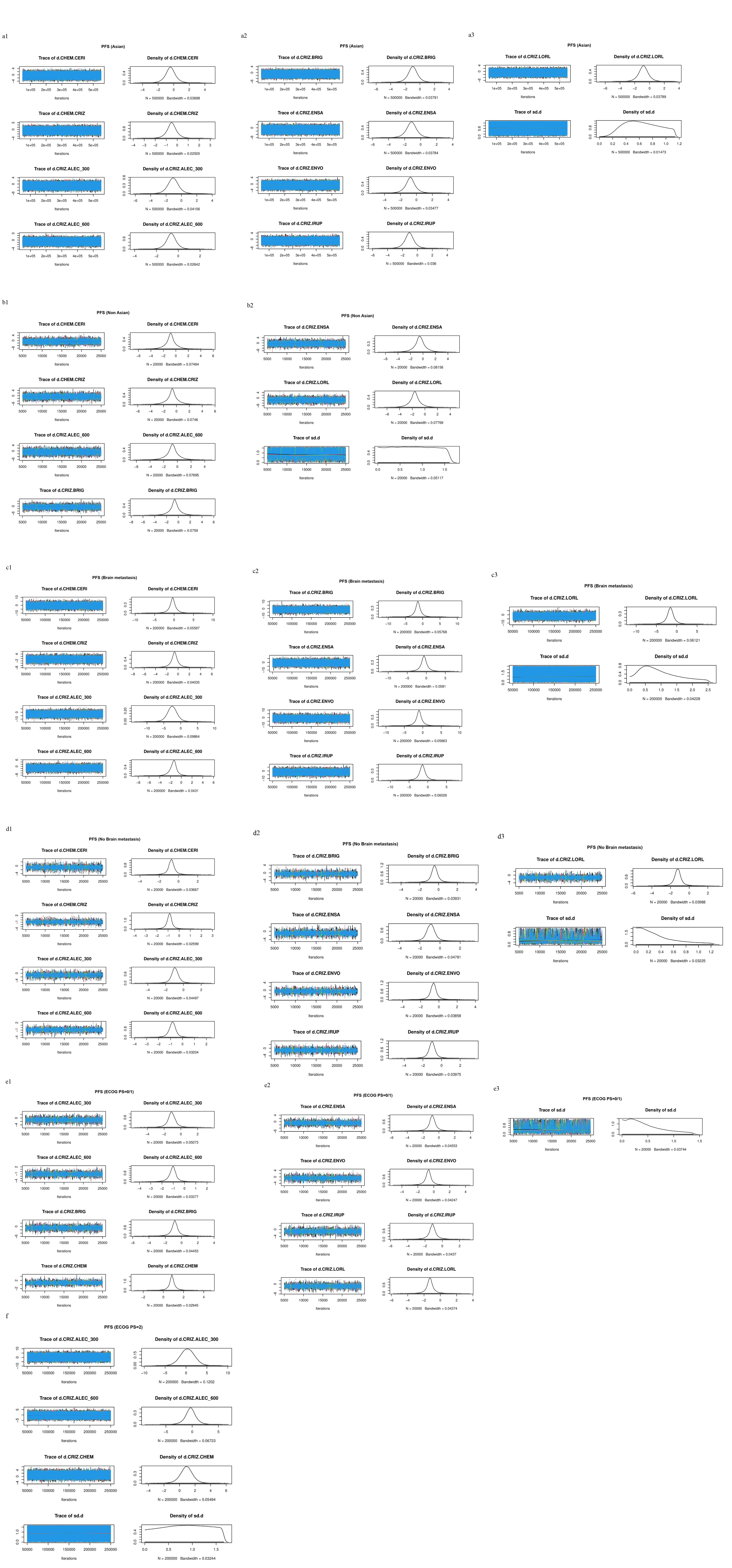


**Fig S17** Trace and Density plot for PFS (ethnicity), PFS (brain metastases) and PFS (ECOG PS).


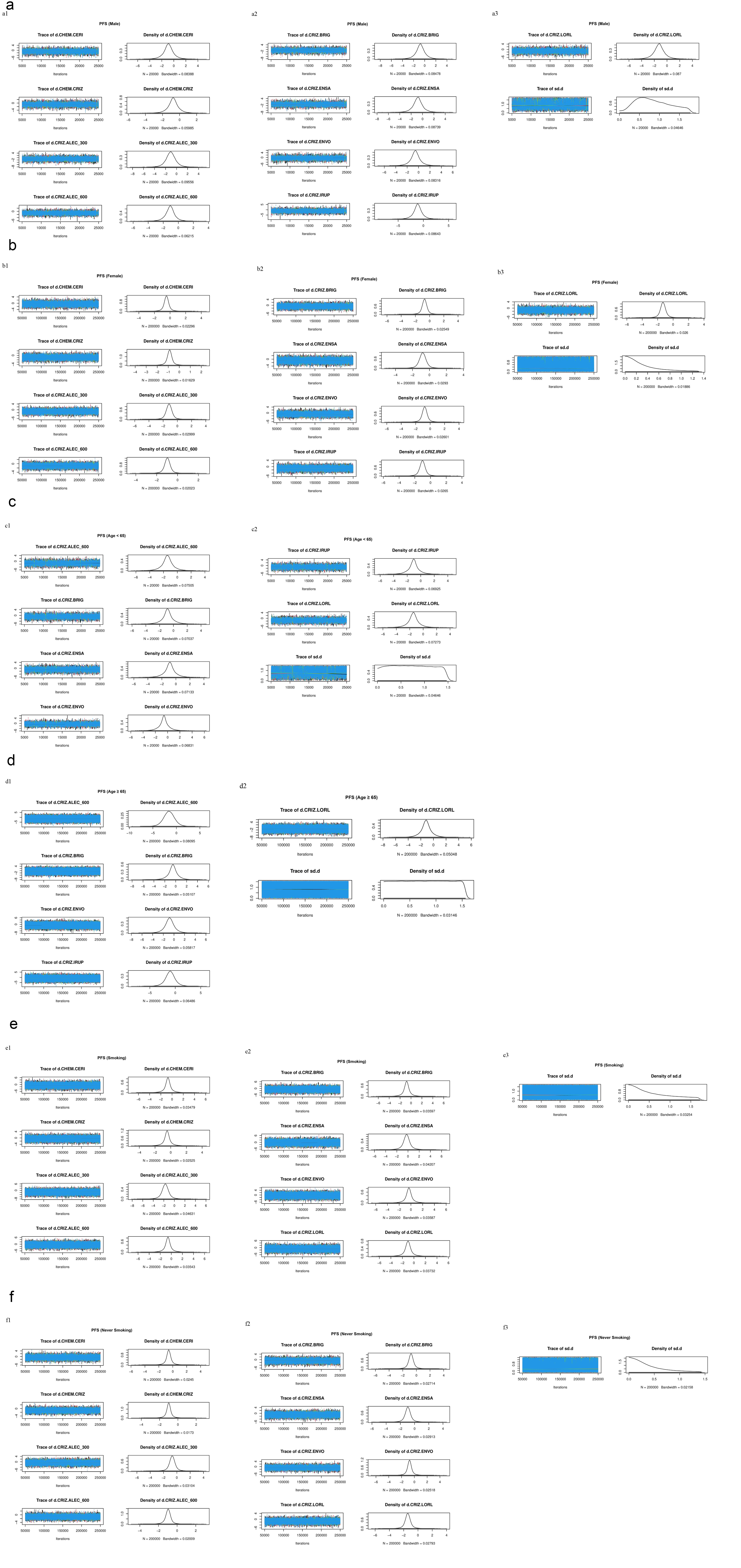


**Fig S18** Trace and Density plot for PFS (sex), PFS (age) and PFS (smoking history).
