# Supplementary material for: Comparison of the efficacy based on clinicopathological characteristics and the safety of first-line treatments for patients with advanced ALK rearrangement non-small cell lung cancer: a network meta-analysis
Source: Front Oncol. 2026 Jan 19;15:1620485. doi: 10.3389/fonc.2025.1620485 (PMC12861906; doi:10.3389/fonc.2025.1620485)
Supplement: Supplementary file 5 [file DataSheet5.docx]

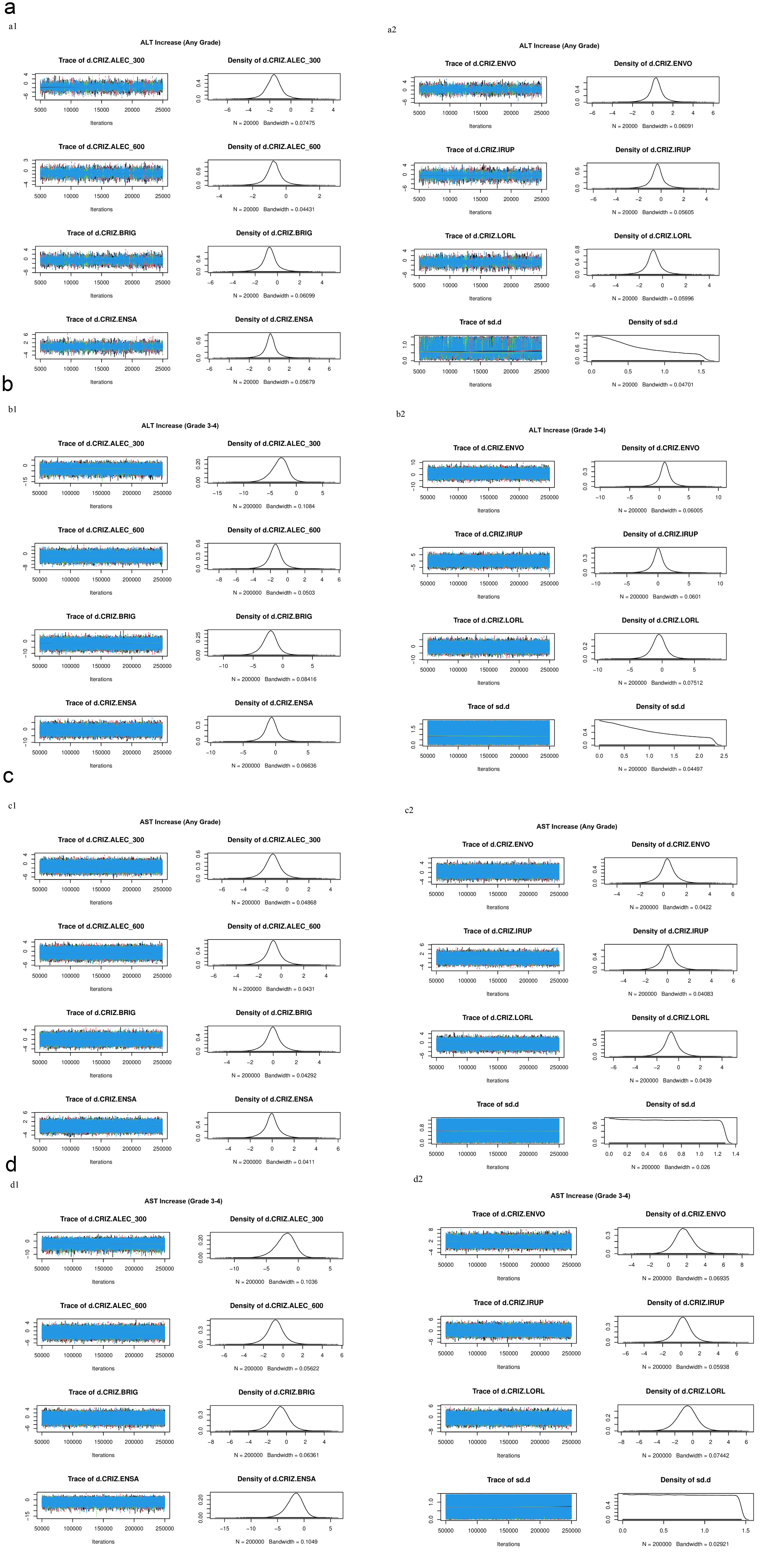


**Fig S19** Trace and Density plot for hepatic AEs (increased ALT/AST).


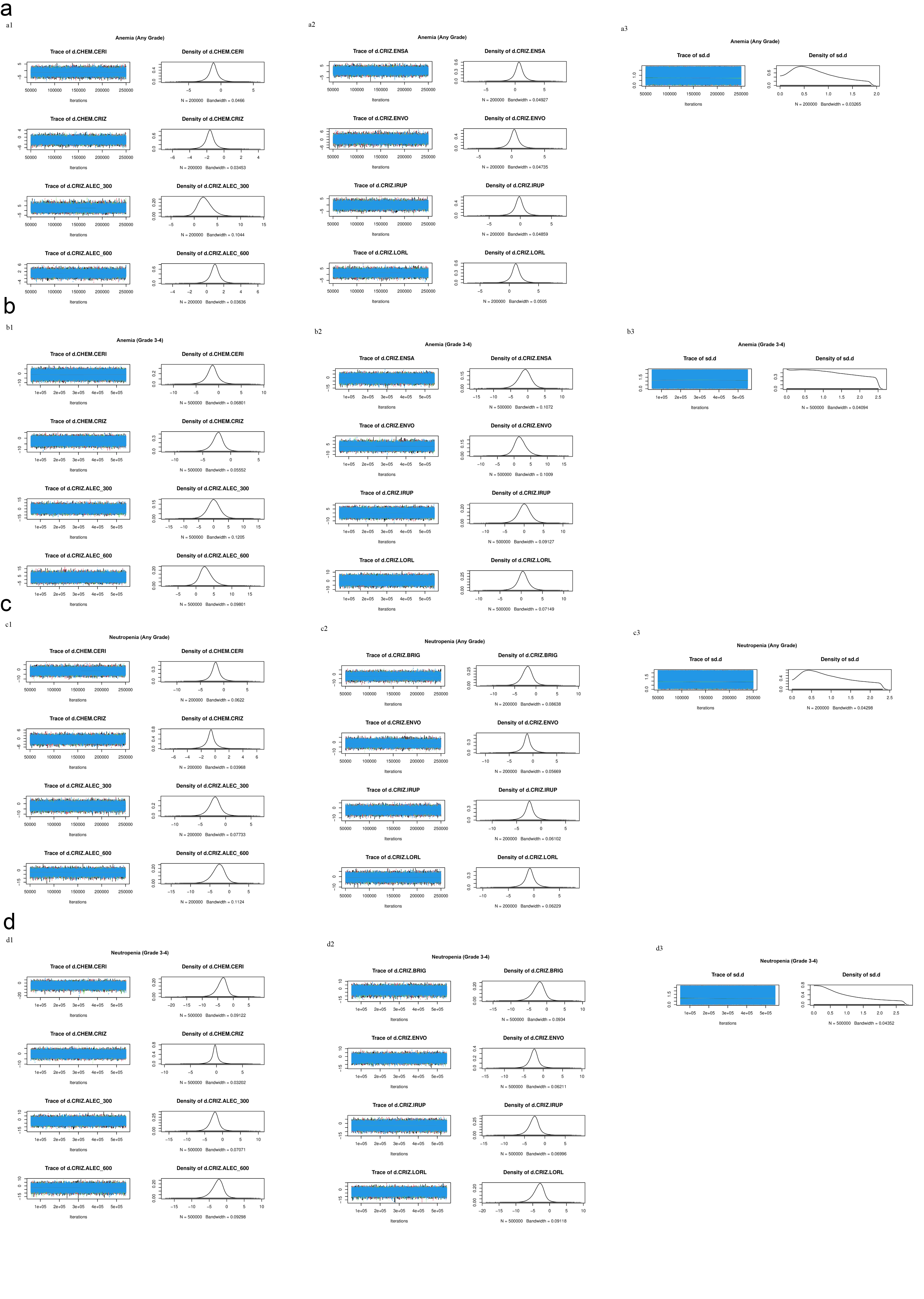


**Fig S20** Trace and Density plot for hematological AEs (anemia/neutropenia).


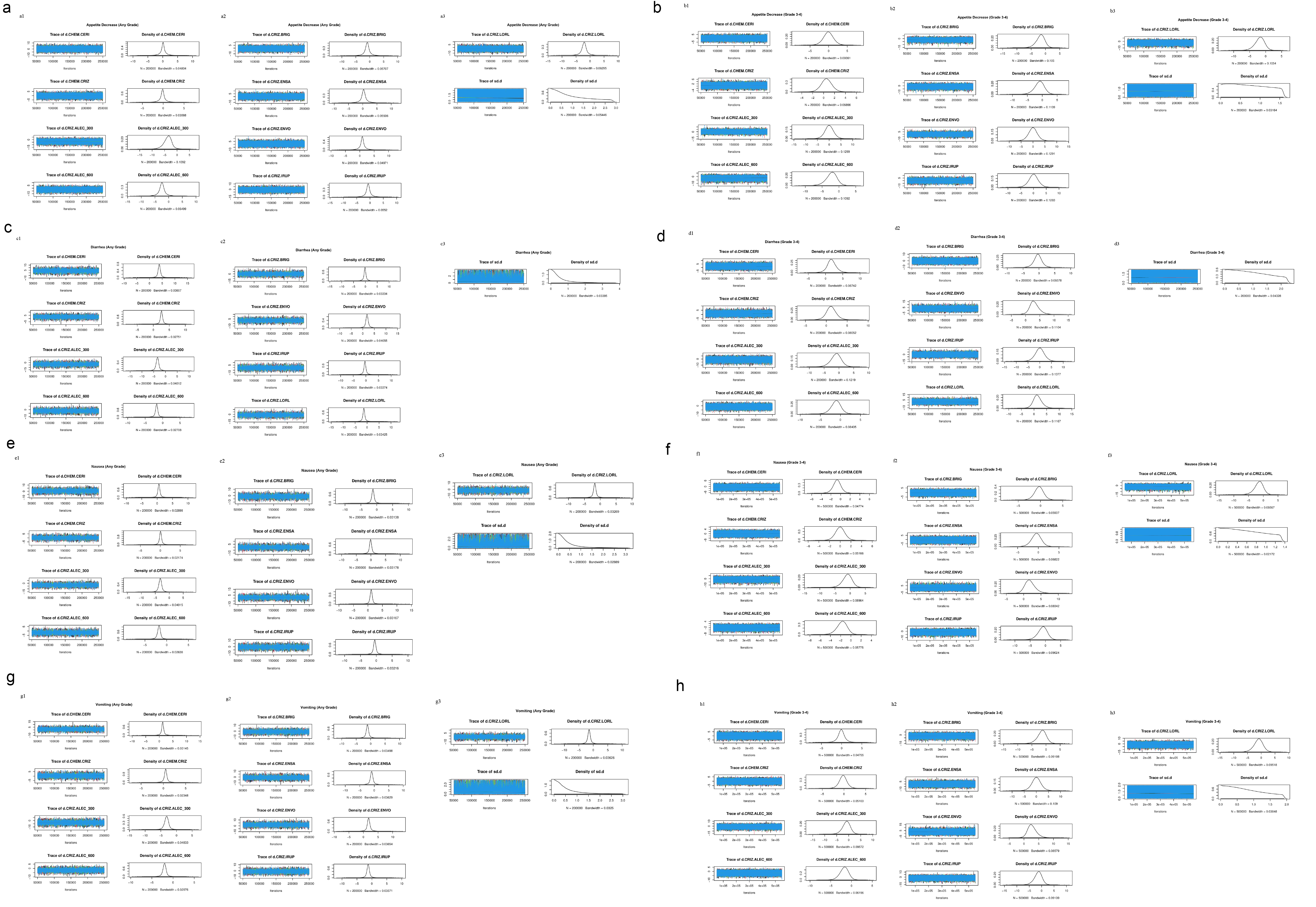


**Fig S21** Trace and Density plot for gastrointestinal AEs (decreased appetite, diarrhea, nausea, vomiting).
